# Defining and reporting adverse events of special interest in comparative maternal vaccine studies: a systematic review

**DOI:** 10.1016/j.jvacx.2024.100464

**Published:** 2024-02-23

**Authors:** Hannah G Davies, Emma V Thorley, Rossul Al-Bahadili, Natalina Sutton, Jessica Burt, Lauren Hookham, Kostas Karampatsas, Philipp Lambach, Flor Muñoz, Clare L Cutland, Saad Omer, Kirsty Le Doare

**Affiliations:** a Centre for Neonatal and Paediatric Infection, Institute of Infection & Immunity, St George’s, University of London, Cranmer Terrace, Tooting, London, United Kingdom; b MRC, UVRI & LSHTM Uganda Research Centre, Entebbe, Uganda; c Makerere University John Hopkins Research Unit, Kampala, Uganda; d World Health Organization, Geneva, Switzerland; e Paediatric Infectious Diseases Department, Baylor College of Medicine, Houston, TX, USA; f Wits African Leadership in Vaccinology Expertise (Wits-Alive), School of Pathology, Faculty of Health Science, University of the Witwatersrand, Johannesburg, South Africa; g O’Donnell School of Public Health, UT Southwestern Medical Center, Texas, USA

**Keywords:** Pharmacovigilance, Vaccines, Pregnancy, Maternal interventions vigilance, Safety

## Abstract

**Introduction:**

The GAIA (Global Alignment on Immunisation Safety Assessment in Pregnancy) consortium was established in 2014 with the aim of creating a standardised, globally coordinated approach to monitoring the safety of vaccines administered in pregnancy. The consortium developed twenty-six standardised definitions for classifying obstetric and infant adverse events. This systematic review sought to evaluate the current state of adverse event reporting in maternal vaccine trials following the publication of the case definitions by GAIA, and the extent to which these case definitions have been adopted in maternal vaccine safety research.

**Methods:**

A comprehensive search of published literature was undertaken to identify maternal vaccine research studies. PubMed, EMBASE, Web of Science, and Cochrane were searched using a combination of MeSH terms and keyword searches to identify observational or interventional studies that examined vaccine safety in pregnant women with a comparator group. A two-reviewer screening process was undertaken, and a narrative synthesis of the results presented.

**Results:**

14,737 titles were identified from database searches, 435 titles were selected as potentially relevant, 256 were excluded, the remaining 116 papers were included. Influenza vaccine was the most studied (25.0%), followed by TDaP (20.7%) and SARS-CoV-2 (12.9%).

Ninety-one studies (78.4%) were conducted in high-income settings. Forty-eight (41.4%) utilised electronic health-records. The majority focused on reporting adverse events of special interest (AESI) in pregnancy (65.0%) alone or in addition to reactogenicity (27.6%). The most frequently reported AESI were preterm birth, small for gestational age and hypertensive disorders. Fewer than 10 studies reported use of GAIA definitions. Gestational age assessment was poorly described; of 39 studies reporting stillbirths 30.8% provided no description of the gestational age threshold.

**Conclusions:**

Low-income settings remain under-represented in comparative maternal vaccine safety research. There has been poor uptake of GAIA case definitions. A lack of harmonisation and standardisation persists limiting comparability of the generated safety data.

## Introduction

Neonatal mortality continues to pose a significant global health challenge, accounting for almost half of all under-5 deaths worldwide [1]. While significant progress has been made in reducing child mortality, neonatal mortality remains a persistent concern, particularly in low- and middle-income countries (LMICs). Maternal vaccination has emerged as a critical intervention with the potential to substantially impact neonatal health outcomes. New vaccines are under development to address key pathogens impacting infant mortality [2]. The potential of this approach has been evidenced by the maternal-neonatal tetanus program in which the well-established tetanus vaccine is one of the key interventions [3]. First implemented in 1989, by 2020, all but 12 targeted countries had reached elimination status [4]. Vaccinations during pregnancy also help safeguard the mother from vaccine-preventable illnesses such as influenza and SARS CoV-2, reducing the risk of severe complications of diseases and associated adverse outcomes [5].

According to the World Health Organization (WHO), Immunisation currently prevents 3.5–5 million deaths every year from diseases like diphtheria, tetanus, pertussis, influenza and measles [6]. However, despite the available evidence of effectiveness and established reassuring safety profiles of vaccines, vaccine hesitancy has emerged as a concerning global phenomenon [7]. The rise in vaccine hesitancy, which has been demonstrated in pregnancy, poses a grave threat to public health, jeopardising the significant progress achieved through immunisation programs. Demonstrating a commitment to vigilance, transparency, and evidence-based decision-making, vaccine safety research builds public trust in vaccines. Such systems provide reassurance that the safety of individuals and communities is a top priority throughout the entire vaccine life cycle, fostering confidence in the benefits of immunisation and ultimately contributing to higher vaccine acceptance rates.

The Brighton Collaboration, an international network of experts established in 2000, has developed standardised case definitions for adverse events following immunisation (AEFI) in order to provide a common language and framework for identifying, classifying, and reporting vaccine-related adverse events. Their goal is to improve the collection and analysis of vaccine safety data through the use of consistent terminology and criteria to enable comparison across different studies, surveillance systems and settings.

The GAIA (Global Alignment on Immunisation safety Assessment in pregnancy) consortium was formed as part of the Brighton Collaboration, to address the specific need for standardised methods for assessing the safety of research vaccines during pregnancy. A systematic review to assess variability in AEFI reporting was conducted as a preliminary step in development of these standardised definitions. This review identified variability in how the presence of AEFIs were determined, how AEFI definitions were applied, and in the ways that AEFIs were reported. Definitions for AEFIs differed in terms of level of detail, boundaries and cut-offs, severity strata and standards used [8]. The GAIA group sought to address these issues through development of standardised maternal, fetal and infant event definitions for the classification of adverse events of special interest (AESI) [9]. These case definitions were developed with the aim of achieving sufficient applicability to be of use in monitoring immunisation safety in pregnancy globally [10]. The selection of these outcomes was prioritised based on recommendations from global experts convened by the World Health Organization in 2014 [10]. The first ten definitions were published in 2016, these included stillbirth, neonatal death, maternal death, congenital anomalies, non-reassuring fetal status, hypertensive disorders in pregnancy, pathways to preterm birth, postpartum haemorrhage, preterm birth and neonatal infections. These were followed by the next twelve definitions in 2017 – congenital microcephaly, fetal growth restriction, antenatal bleeding, dysfunctional labor, gestational diabetes, spontaneous abortion, ectopic pregnancy, neonatal encephalopathy, failure to thrive, low birthweight, respiratory distress and small for gestational age. A further four which include chorioamnionitis, postpartum endometritis, neonatal seizures and neurodevelopmental delay were published in 2019 [11]. The definitions categorise the outcomes into levels of diagnostic certainty (1–3), with greatest specificity at the highest level (level 1) and increasing sensitivity as you progress through the levels, whilst still maintaining an acceptable specificity. They were developed in this way to accommodate the resources and diagnostic capabilities available in different locations.

This updated review aimed to assess the status of maternal vaccine AEFI reporting in comparative clinical vaccine trials following publication of the standardised case definitions by GAIA. We assessed the extent to which these guidelines and case definitions have been adopted in maternal vaccine safety research since their publication seven years ago.

## Methods

The study objectives were to describe the study characteristics, types of vaccines researched in pregnancy, the frequency of reported adverse event outcomes, classification, or provision of definitions for the adverse event outcome and use of Brighton Collaboration GAIA definitions. We also aimed to describe the consistency observed in AEFI reporting, variability in definitions utilised and the adverse event data collection methodologies. The study was registered on the international prospective register of systematic reviews (PROSPERO) CRD42021253680.

### Eligibility criteria

Studies, whether interventional or observational, that involved administration of any vaccine(s) to pregnant women of any age were included in the review. Studies that did not include pregnant women, either as the main participants or as an at-risk group were excluded. Studies making any relevant comparison of vaccines against a control, such as placebo, alterative vaccine, unexposed or untreated group, were included, studies that did not include a comparator were excluded. Acceptable outcome measures included intervention efficacy, effectiveness, or safety. Studies that did not evaluate vaccine safety as a primary outcome were included if maternal, or neonatal safety or adverse event data were presented. The study setting had no impact on inclusion. Studies conducted in any language other than English were excluded as were unpublished studies. Studies were limited to those published between the date of the previous review by Fulton et al [8] (2014) and the date of the search.

### Search strategy

A systematic search of published literature potentially containing data on maternal and neonatal adverse events following maternal immunisation was conducted. All published comparative maternal immunisation studies (randomised controlled trials and observational studies), identified via searches of PubMed, EMBASE, Web of Science, and the Cochrane Database using a combination of medical subject headings (MeSH) terms and keyword searches were included. The search strategy used for this review was derived from prior work by Fulton et al [8]. Supplementary Tables 1, 2, 3, 4 outlines the search strategies in the four databases searched. Results were limited to English language publications between 2014 and December 31st, 2022.

### Selection & data extraction

**Fig. 1:**
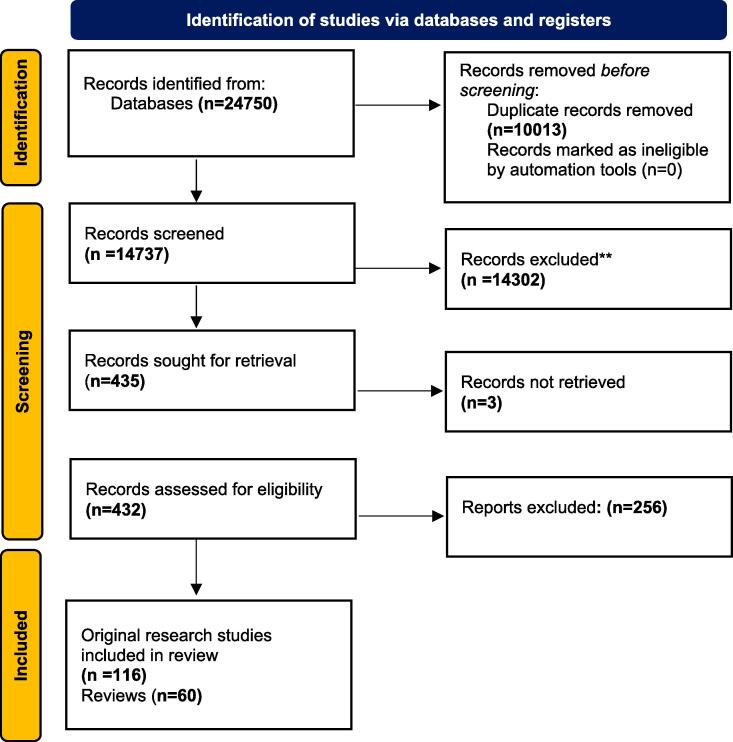
PRISMA Flow Diagram.

A two-reviewer system was adopted for the entire review process. Results of the database searches were imported into Mendeley Desktop and automated deduplication followed by manual deduplication was undertaken. Two reviewers (HGD + KK, LH or ET) screened all titles and abstracts for eligibility. Articles were excluded if their titles and abstracts were clearly unrelated to the criteria of this review. Full texts of all eligible studies were retrieved and reviewed by two reviewers (HGD, KK or ET), studies for which full texts were inaccessible were discarded. The rationale for study exclusion was recorded as part of the screening process. Consensus or recourse to a third review reviewer (KLD) occurred in the case of uncertainty during the screening process with regards to inclusion/exclusion. An adapted PRISMA (Preferred Reporting Items for Systematic Reviews and Meta-Analyses) flow-chart of study selection was completed (Fig. 1 & supplementary Fig. 1) [12].

The objective of this review was to determine the variance in AEFI definitions across all comparative maternal immunisation studies within the prespecified dates, irrespective of study design, rigor, outcome, or potential bias. A methodological study quality assessment (Grading of Recommendations Assessment, Development and Evaluation (GRADE) analysis) was, therefore, not needed. Studies included were not assessed for, nor ranked based on limitations in design or possible bias. A narrative synthesis of the evidence on AEFI reporting in maternal vaccine studies was undertaken. All data from studies meeting the inclusion criteria were extracted into an Excel workbook for analysis. Advanced data analysis, when required, was conducted in Stata version 18.0.

## Results

### Study selection & characteristics

14,737 titles were identified from electronic searches, 435 titles were selected as potentially relevant, 432 were retrieved and assessed for eligibility. Following assessment, 256 papers were excluded, and the remaining 176 papers were included in the review (Fig. 1). 116 of these were original research papers (Table 1) and 60 review papers (Supplementary Table 5).

**Table 1:** Description of all included studies (excluding review papers).

ID	Author	Year of publication	Continent	Vaccine	Income status	Primary study outcome
	Influenza					
1	Ma et al. [34]	2014	Asia	Influenza	UMI	Safety outcomes (AEFI & pregnancy outcomes).
2	Legge et al. [35]	2014	North America	Influenza	HI	Influenza vaccination rates among pregnant women, maternal factors as predictors of influenza vaccination status and the association between maternal influenza vaccination and neonatal outcomes.
3	Ahrens et al. [36]	2014	North America	Influenza	HI	Associations between seasonal influenza vaccination during pregnancy and the risks of preterm delivery and small for gestational age.
4	Tapia et al. [37]	2016	Africa	Influenza	LI	Efficacy of maternal immunisation for prevention of laboratory-confirmed influenza in their infants
5	Regan et al. [38]	2016	Australia	Influenza	HI	Relative risk of stillbirth among vaccinated and unvaccinated pregnant women.
6	Chambers et al. [39]	2016	North America	Influenza	HI	The fetal risk and relative safety of the seasonal influenza vaccine
7	Louik et al. [40]	2016	North America	Influenza	HI	Risks for preterm delivery (PTD) and specific birth defects following vaccination in the 2011–12 through 2013–14 influenza seasons.
8	Olsen et al. [41]	2016	Asia	Influenza	LMI	The effect of influenza vaccine on birth outcomes.
9	Steinhoff et al. [42]	2017	Asia	Influenza	LMI	Incidence of laboratory-confirmed influenza illness in the infant, incidence of influenza-like illness in the mothers during pregnancy post-partum, and incidence of low birthweight.
10	Kozuki et al. [43]	2017	Asia	Influenza	LMI	Birth weight and pregnancy length among participants enrolled in the Nepal Mothers’ GiftTrial.
11	Kharbanda et al. [20]	2017	North America	Influenza	HI	Presence of 1 or more prespecified major structural birth defects.
12	Kotinov et al. [44]	2018	Europe/Asia	Influenza	UMI	Comparative immunogenicity analysis of different subunit influenza vaccines in pregnant women.
13	Arriola et al. [45]	2017	Central America	Influenza	LMI	Adverse birth outcomes in neonates born to mothers vaccinated against influenza.
14	Asavapiriyanont et al. [46]	2018	Asia	Influenza	UMI	Frequency of AEFIs after influenza vaccination and birth outcomes.
15	McHugh et al. [47]	2018	Australia	Influenza	HI	Ascertain seasonal IIV uptake in pregnancy amongst a cohort of remote-living Aboriginal women in the NT.
16	Getahun et al. [48]	2019	North America	Influenza	HI	Safety of seasonal influenza vaccination in pregnant women from a large, ethnically diverse, patient population.
17	Donahue et al. [49]	2019	North America	Influenza	HI	To determine if receipt of IIV was associated with SAB among women who had and had not been administered influenza vaccine the previous season.
18	Simões et al. [50]	2019	Africa	Influenza	UMI	Laboratory-confirmed infant and maternal influenza in HIV- uninfected mother-infant dyads
19	Singh et al. [51]	2019	Asia	Influenza	LMI	To see the efficacy of influenza vaccine on pregnant women and their newborn up to 6 months.
20	Nunes et al. [52]	2020	Africa	Influenza	UMI	Immunogenicity of a double dose and two single doses of inactivated influenza vaccine compared with a single dose of inactivated influenza vaccine in pregnant women living with HIV to each of the three vaccine strains, and relative safety of the three dosing schedules.
21	Munoz et al. [21]	2020	North America	Influenza	HI	Injection site and systemic reactions in the pregnant women for 7 days following receipt of the study vaccine; vaccine-associated maternal AEs and maternal and infant SAEs for the duration of study participation; and pregnancy outcomes, including maternal and neonatal complications during pregnancy and at time of delivery.
22	Avalos et al. [53]	2020	North America	Influenza	HI	Whether receipt of trivalent inactivated influenza vaccine (IIV3) during pregnancy impacts 6-month infant development.
23	Ohfuji et al. [54]	2020	Asia	Influenza	HI	Adverse birth outcomes (including miscarriage, stillbirth, preterm birth, low birth weight, and congenital malformation) between vaccinated and unvaccinated pregnant women in Japan.
24	Vesikari et al. [27]	2020	Europe	Influenza	HI	Immune response and safety of one dose of IIV4 (VaxigripTetra, Sanofi Pasteur) or IIV3 (Vaxigrip, Sanofi Pasteur) 21 days after vaccination in pregnant women.
25	Peppa et al. [55]	2020	Europe	Influenza	HI	Assess vaccine safety in relation to major congenital malformations (MCMs)
26	Mohammed et al. [56]	2020	Australia	Influenza	HI	Safety and protective effect of maternal influenza vaccination on pregnancy and birth outcomes.
27	Speake et al. [57]	2021	Australia	Influenza	HI	Risk of adverse maternal and foetal outcomes associated with inactivated influenza vaccination in first trimester of pregnancy
28	McMorrow et al. [58]	2021	Africa	Influenza	UMI	To assess the effect of maternal antenatal influenza vaccination on birth outcomes.
29	Palmsten et al. [59]	2022	North America	Influenza	HI	The association between early pregnancy influenza vaccination and specific CHDs, including those not previously examined in the literature.

	Tetanus, diphtheria & acellular pertussis (TDaP)					
30	Donegan et al. [60]	2014	Europe	TDaP	HI	Adverse events identified from clinical diagnoses during pregnancy.
31	Kharbanda et al. [61]	2014	North America	TdaP	HI	To evaluate whether maternal Tdap vaccination during pregnancy is associated with increased risks of adverse obstetric events or adverse birth outcomes.
32	Morgan et al. [62]	2015	North America	TDaP	HI	Pregnancy outcomes of women who received tetanus, diphtheria, and acellular pertussis (Tdap) vaccination at or after 32 weeks of gestation. Additionally, to compare pregnancy outcomes in women who were administered Tdap vaccine in consecutive pregnancies within a 5-year timespan.
33	Sukumaran et al. [63]	2015	North America	Tdap	HI	Whether receipt of Tdap vaccine during pregnancy administered in close intervals from prior tetanus-containing vaccinations is associated with acute adverse events in mothers and adverse birth outcomes in neonates.
34	Maertens et al. [64]	2016	Europe	TDaP	HI	Differences in antibody titers at several time points.
35	Hoang et al. [65]	2016	Asia	TDaP	LMI	Amount of transferred maternal antibodies and the possible interference of the vaccine with humoral immune responses in the infants.
36	Berenson et al. [66]	2016	North America	TDaP	HI	Maternal and infant outcomes between women who did and did not receive the Tdap vaccine during pregnancy.
37	Kharbanda et al. [67]	2016	North America	TDaP	HI	Estimates of Tdap coverage during pregnancy among insured women within the Vaccine Safety Datalink.
38	DeSilva et al. [68]	2017	North America	TDaP	HI	Association between maternal Tdap and chorioamnionitis and examined risks for specific infant morbidities following maternal Tdap vaccination
39	Villareal Perez [69]	2017	North America	TDaP	HI	Immunogenicity and interference of maternal antibodies.
40	Layton et al. [70]	2017	North America	TDaP	HI	Whether prenatal Tdap immunization was associated with adverse birth outcomes, and to evaluate the effect of timing of Tdap administration on these outcomes.
41	Wanlapakorn et al. [71]	2018	Asia	TDaP	UMI	Reactogenicity profile of Tdap vaccine in a randomized controlled clinical trial involving Tdap-vaccinated Thai mothers, concentrations of B. pertussis-specific antibodies in paired maternal and umbilical cord sera and adverse events and pregnancy outcomes when multiple tetanus-containing vaccines are administered.
42	Becerra-Culqui et al. [72]	2018	North America	TDaP	HI	Association between prenatal tetanus, diphtheria, acellular pertussis (Tdap) vaccination and autism spectrum disorder (ASD) risk in offspring.
43	Griffin et al. [16]	2018	Oceania	Tdap	HI	The safety of Tdap vaccine administered to pregnant women in 2013.
44	Halperin et al. [73]	2018	North America	Tdap	HI	Safety and immunogenicity of Tdap during pregnancy and the effect on the infant’s immune response to primary vaccination at 2, 4, and 6 months and booster vaccination at 12 months of age.
45	Fortner et al. [74]	2018	North America	Tdap	HI	Compare local injection-site and systemic reactions and serologic response following Tdap in (1) pregnant and nonpregnant women and (2) pregnant women by self-reported prior Tdap receipt.
46	Barug et al. [75]	2019	Europe	TDaP	HI	Serum IgG pertussis toxin antibody concentrations at age 3 months.
47	Sancovski et al. [76]	2019	South America	TDaP	UMI	Compare the risk of gestational diabetes, pregnancy-related hypertension, and pregnancy hemorrhage in women from exposed and unexposed cohorts and the risk of preterm birth and small for gestational age in their neonates.
48	Petousis-Harris et al. [15]	2019	Oceania	TDaP	HI	Difference in birth and hospital-related outcomes of infants with and without fetal exposure to Tdap.
49	Perrett et al. [26]	2020	Mixed	TDaP	HI	The amount of maternally transferred pertussis antibodies in cord blood of Tdap-vaccinated mothers and placebo-vaccinated mothers.
50	Becerra-Culqui et al. [77]	2020	North America	TDaP	HI	ADHD diagnosis following TDaP vaccine.
51	Hall et al. [78]	2020	North America	TDaP	HI	Adverse pregnancy and infant outcomes
52	Kerr et al. [79]	2020	North America	Tdap	HI	Analysis of Tdap vaccine exposure during pregnancy and the risk for specific congenital malformations.
53	Mohammed et al. [19]	2021	Australia	TDaP	HI	Safety of maternal pertussis vaccination on pregnancy and birth outcomes.

	SARS-CoV-2					
54	Shimabukuro et al. [80]	2021	North America	SARS-CoV-2	HI	Local and systemic adverse reactions to the vaccine amongst pregnant women.
55	Gray et al. [81]	2021	North America	SARS-CoV-2	HI	Immunogenicity and reactogenicity of coronavirus disease 2019 messenger RNA vaccination in pregnant and lactating women compared with: (1) nonpregnant controls and (2) natural coronavirus disease 2019 infection in pregnancy
56	Shimabukuro et al. [82]	2021	North America	SARS-CoV-2	HI	mRNA Covid-19 vaccine safety in pregnant persons from three U.S. vaccine safety monitoring systems.
57	Sadarangani et al. [83]	2022	North America	SARS-CoV-2	HI	Rates of health events in vaccinated pregnant females and vaccinated non-pregnant females of the same age and (2) vaccinated and unvaccinated (control) pregnant females.
58	Nevo et al. [84]	2022	Asia	SARS-CoV-2	HI	Dynamics of anti-SARS-CoV-2 antibody levels following SARS-CoV-2 infection during pregnancy and maternal and neonatal impact of a single post-infection boosting dose of the Pfizer BNT162b2 mRNA vaccine.
59	Blakeway et al. [85]	2022	Europe	SARS-CoV-2	HI	Immunogenicity and reactogenicity of COVID-19 vaccines in pregnant compared with non-pregnant women receiving two doses.
60	Favre et al. [86]	2022	Europe	SARS-CoV-2	HI	Early adverse events in pregnant women, as well as perinatal outcomes after exposure to COVID-19 vaccine any time during pregnancy
61	Kugelman et al. [87]	2022	Asia	SARS-CoV-2	HI	Risk of adverse perinatal outcome among vaccinated and unvaccinated pregnant women from a single centre
62	Hui et al. [88]	2022	Australia	SARS-CoV-2	HI	Perinatal outcomes associated with vaccination in pregnancy, including preterm birth, stillbirth, and congenital anomalies.
63	Calvert et al. [13]	2022	Europe	SARS-CoV-2	HI	Association between COVID-19 vaccination, miscarriage and ectopic pregnancy.
64	Kachikis et al. [89]	2022	North America	SARS-CoV-2	HI	Reactions to the COVID-19 vaccine booster doses and vaccine experiences among pregnant and lactating individuals.
65	Toussia-Cohen et al. [90]	2022	Asia	SARS-CoV-2	HI	Vaccine-induced immunity and adverse events associated with the third (booster) dose of the BNT162b2 vaccine among pregnant women compared with pregnant women who received the first and second dose of the vaccine.
66	Rottenstreich et al. [91]	2022	Asia	SARS-CoV-2	HI	Impact of the third Covid-19 booster dose (Pfizer-BioNTech BNT162b2) on maternal and neonatal outcomes.
67	Fell et al. [92]	2022	North America	SARS-CoV-2	HI	Association of the vaccine with risk of preterm birth (including spontaneous preterm birth and very preterm birth), small for gestational age at birth, or stillbirth.
68	Fell et al. [93]	2022	North America	SARS-CoV-2	HI	Association between vaccination and postpartum hemorrhage, chorioamnionitis, caesarean delivery (overall and emergency caesarean delivery), admission to neonatal intensive care unit, and low newborn Apgar score.

	H1N1					
69	Nordin et al. [94]	2014	North America	H1N1	HI	Risks of acute adverse events and maternal complications within 6 weeks of receiving MIV.
70	Van der Maas et al. [95]	2014	Europe	H1N1	HI	Safety of vaccination with Focetria during the second and third trimesters of pregnancy. Impact of the vaccination on pregnancy outcomes and growth.
71	Beau et al. [96]	2014	Europe	H1N1	HI	Adverse pregnancy outcomes for pregnant women exposed or not exposed to an A/H1N1 vaccine
72	Cleary et al. [97]	2014	Europe	H1N1	HI	Describe the uptake and determinants of 2009A/H1N1 influenza vaccination in pregnant women during the pandemic.
73	Trotta et al. [98]	2014	Europe	H1N1	HI	Risk of maternal, fetal, and neonatal outcomes associated with the administration of an MF59 adjuvanted A/H1N1 vaccine during pregnancy.
74	Huang et al. [99]	2014	Asia	H1N1	HI	Association between maternal H1N1 vaccination and spontaneous abortion (SAB) or adverse fetal outcomes in Taiwan.
75	Fabiani et al. [100]	2015	Europe	H1N1	HI	The risk of adverse maternal, fetal and neonatal outcomes associated with the administration of the MF59-adjuvanted A/H1N1pdm09 influenza vaccine in pregnant women and newborns.
76	Coenders et al. [101]	2015	Europe	H1N1	HI	Evaluate a possible association between the H1N1/09 vaccinations and the occurrence of PE and/or IUGR
77	Baum et al. [102]	2015	Europe	H1N1	HI	Safety of the AS03 adjuvanted pandemic influenza vaccine given during pregnancy.
78	Ludvigsson et al. [103]	2016	Europe	H1N1	HI	Risk of any congenital malformation among offspring of mothers exposed to Pandemrix.
79	Conlin et al. [104]	2018	North America	H1N1	HI	Birth defects in children of women vaccinated with H1N1 vaccine versus seasonal influenza vaccine.
80	Ludvigsson et al. [105]	2020	Europe	H1N1	HI	Whether maternal influenza A(H1N1) pdm09 vaccination during pregnancy was associated with increased risk for ASD in offspring.

	Human Papillomavirus					
81	Angelo et al. [106]	2014	Europe	HPV	HI	Evaluate the safety of the human papillomavirus (HPV)-16/18-AS04-adjuvanted vaccine.
82	Baril et al. [107]	2015	Europe	HPV-16/18	HI	Risk of spontaneous abortion within a cohort of vaccinated women.
83	Panagiotou et al. [108]	2015	North America	HPV	UMI	Effects of HPV vaccine on the risk of miscarriage for pregnancies conceived less than 90 days from vaccination.
84	Lipkind et al. [109]	2017	North America	HPV	HI	Whether inadvertent 4vHPV exposures in the periconceptional period, or during pregnancy, were associated with increased risks for adverse maternal or infant outcomes.
85	Scheller et al. [110]	2017	Europe	HPV	HI	Birth outcomes in women exposed to HPV vaccine during pregnancy.
86	Kharbanda et al. [111]	2019	North America	HPV	HI	Risks for spontaneous abortion after 4vHPV vaccination during pregnancy or *peri*-pregnancy within the Vaccine Safety Datalink.
87	Faber et al. [112]	2018	Europe	HPV	HI	The association between HPV vaccination during pregnancy and subsequent risk of spontaneous abortion, stillbirth, and one-year infant mortality.
88	Bukowinski et al. [113]	2020	North America	HPV	HI	Evaluate how inadvertent exposure to 4vHPV in pregnancy among active-duty US military women is associated with maternal and infant health outcomes.
89	Kharbanda et al. [114]	2021	North America	HPV	HI	Associations between 9vHPV vaccine exposures during pregnancy or *peri*-pregnancy and selected pregnancy and birth outcomes.

	Influenza & TDaP					
90	Sukumaran et al. [159]	2015	North America	TDaP & influenza	HI	Whether there was an increased risk of medically attended acute events or adverse birth outcomes when Tdap and influenza vaccines are administered concomitantly during pregnancy
91	Regan et al. [115]	2016	Australia	Influenza & TDaP	HI	Reactogenicity of seasonal inactivated trivalent influenza vaccine (TIV) and diphtheria-tetanus-acellular pertussis (TdaP) vaccines administered in a cohort of pregnant women.
92	Zerbo et al. [117]	2017	North America	Influenza (TDaP as secondary exposure)	HI	Association between influenza vaccination during pregnancy and birth outcomes.
93	Panagiotakopoulos et al. [18]	2020	North America	Influenza & TDaP	HI	The association between vaccination during pregnancy and risk of stillbirth.

	Cholera					
94	Grout et al. [118]	2015	Africa	Cholera	LI	Difference in pregnancy outcomes between women who exposed their fetus to OCV and those who did not.
95	Khan et al. [119]	2017	Asia	OCV	LMI	Adverse fetal outcomes of miscarriage, stillbirth, and congenital anomaly.
96	Ali et al. [120]	2017	Africa	Oral cholera vaccine	LI	Pregnancy loss (spontaneous miscarriage or stillbirth).
97	Khan et al. [121]	2019	Asia	OCV	LMI	Pregnancy loss (spontaneous miscarriage or stillbirth), and secondary endpoints were preterm delivery and low birth weight.

	Influenza & H1N1					
98	Vasquez-Benitez [122]	2016	North America	H1N1 & influenza	HI	Preterm birth and small for gestational age at birth.
99	Eaton et al. [123]	2018	North America	H1N1 & TIV	HI	Safety of H1N1 vaccine compared with TIV vaccine administered during all three trimesters by evaluating birth outcomes following immunization of pregnant women.
100	Munoz et al. [22]	2018	North America	Influenza & H1N1	HI	Safety and immunogenicity of two licensed seasonal trivalent IIVs (IIV3) in pregnant women.

	Group B Streptococcus					
101	Donders et al. [124]	2016	Multiple	GBS	HI	Placental transfer of GBS-specific antibodies to newborns born to pregnant women administered an investigational trivalent CRM197 or a placebo.
102	Madhi et al. [125]	2016	Africa	GBS	UMI	Selection of one vaccine dose (either 0·5 μg, 2·5 μg, or 5·0 μg) based on analysis of serotype-specific antibody responses at delivery (+72 h.
103	Swamy et al. 24	2020	North America	GBS	HI	Reactogenicity and safety of the trivalent GBS vaccine in pregnant women, and safety in their infants.
	Pneumococcus					
104	Binks et al. [126]	2015	Australia	Pneumococcal	HI	Prevalence of middle ear disease; and nasopharyngeal carriage of 23vPPV-type pneumococci.
105	McHugh et al. [127]	2020	Australia	Pneumococcal	HI	Re-analysis of trial safety-data taking into account immortal time bias.
106	Weinberg et al. [25]	2021	South America	Pneumococcal	UMI	Safety of PCV-10 and PPV-23 with placebo administered in pregnancy - incidence of adverse events of grade 3 or higher in mothers and in neonates and the incidence of adverse events of grade 3 or higher judged to be possibly related to the study treatment.
	Others					
107	Wak et al. 128	2015	Africa	Meningococcal Group A	LMI	Rates of prespecified events were compared between pregnant women who received PsA-TT and the 2 control groups
108	Conlin et al. [129]	2017	North America	Anthrax	HI	To determine if inadvertent AVA vaccination during pregnancy is significantly associated with risk of birth defects.
109	Skipetrova et al. [14]	2018	Mixed	Dengue	Mixed	Pregnancy outcomes documented from the inadvertent vaccination of women in early pregnancy during the clinical development of CYD-TDV.
110	Groom et al. [130]	2018	North America	Hepatitis B	HI	Frequency of maternal Hepatitis B vaccine receipt among pregnant women and association between maternal vaccination and pre-specified maternal and infant safety outcomes.
111	Munoz et al. [17]	2019	North America	RSV	HI	Safety of RSV F vaccine in pregnant women and their infants inclusive of at least 1 RSV season.
112	Groom et al. [131]	2019	North America	Hepatitis A	HI	Association between vaccination with HepA during pregnancy and pre-specified maternal and infant safety outcomes among women with live births.
113	Madhi et al. [23]	2020	Multiple	RSV	Mixed	Efficacy of maternal immunization with RSV F protein vaccine for the protection of infants against RSV-associated, medically significant lower respiratory tract infection.
114	Khodr et al. 132	2020	North America	Japanese Encephalitis	HI	Adverse pregnancy and infant health outcomes in relation to JE vaccination during pregnancy.
115	Hall et al. [133]	2020	North America	Yellow Fever	HI	Adverse pregnancy or infant outcomes.
116	Willis et al. [134]	2022	North America	Varicella	HI	Association between vaccination and congenital varicella syndrome.

Influenza vaccine was the most investigated vaccine (25.0%), followed by tetanus, diphtheria, acellular pertussis (TDaP) (20.7%), SARS-CoV-2 (12.9%) and the pandemic influenza vaccine H1N1 (10.3%) (Table 3). Other vaccines investigated included Human Papillomavirus (HPV), Cholera, Group B Streptococcus, and Pneumococcus (Table 2).

**Table 2:** Summary characteristics of included studies.

	Number of Papers
Included Publications by vaccine	
Influenza	29 (25.0)
Tetanus, Diphtheria, acellular Pertussis	24 (20.7)
SARS-CoV-2	15 (12.9)
H1N1	12 (10.3)
Human Papillomavirus	9 (7.8)
Influenza & TDaP	4 (3.4)
Cholera	4 (3.4)
Influenza & H1N1	3 (2.6)
Group B Streptococcus	3 (2.6)
Pneumococcus	3* (2.6)
Respiratory Syncytial Virus	2 (1.7)
Dengue	1 (0.86)
Hepatitis A	1 (0.86)
Hepatitis B	1 (0.86)
Japanese Encephalitis	1 (0.86)
Anthrax	1 (0.86)
Meningococcal A	1 (0.86)
Varicella	1 (0.86)
Yellow Fever	1 (0.86)
Included Publications by study design	
Randomised Controlled Trials	24 (20.7)
Prospective Cohort Studies	23 (19.8)
Retrospective Cohort Studies	59 (50.9)
Case-Control	7 (6.0)
Mixed prospective & retrospective cohort	1 (0.86)
Pooled analysis of clinical trials	1 (0.86)
Long-term follow-up of RCT participants & independent unvaccinated cohort	1 (0.86)
Included Publications by location income	
Low-Income	3 (2.6)
Lower-Middle Income	9 (7.8)
Upper Middle-Income	11 (9.5)
High-Income	91 (78.4)
Mixed	2 (1.7)
Included Publications by adverse event outcome type	
Reactogenicity outcomes	8 (6.9)
Adverse events of special interest	76 (65.5)
Reactogenicity & adverse events of special interest	32 (27.6)

*3 manuscripts reporting from 2 studies.

**Table 3:** Numbers of studies reporting GAIA adverse outcomes and frequency of the outcomes reported in vaccine recipients in included studies.

Outcome	Focus	Definition Publication (Year)	Studies (n)	Reported cases (n)
Hypertensive disorders in pregnancy [135]	Maternal	2016	34	19,430
Chorioamnionitis [136]	Maternal	2019	18	18,273
Pathways to preterm birth [137]	Maternal	2016	21	12,630
Postpartum haemorrhage [138]	Maternal	2016	14	7101
Gestational diabetes [139]	Maternal	2017	18	5038
Antenatal bleeding [140]	Maternal	2017	10	2609
Ectopic Pregnancy [141]	Maternal	2017	3	126
Vaginal bleeding/haemorrhage (antepartum/post-partum)	Maternal	NA#	6	29
Dysfunctional labor [142]	Maternal	2017	3	22
Postpartum endometritis [143]	Maternal	2019	1	9
Maternal death [144]	Maternal	2016	6	7
Spontaneous abortion [141]	Fetus	2017	32	4059
Non-reassuring fetal status [145]	Fetus	2016	5	3075
Stillbirth [146]	Fetus	2016	39	2083
Fetal growth restriction [147]	Fetus	2017	9	1894
IUFD (Spontaneous abortions & stillbirths)	Fetus	NA#	13	171
Small for gestational age [148]	Neonate	2017	44	30,929
Preterm birth [149]	Neonate	2016	69	28,828
Congenital anomalies [150]	Neonate	2016	43	13,466
Low birthweight [151]	Neonate	2017	35	10,075
Respiratory distress [152]	Neonate	2017	6	7853
Neonatal infections [153]	Neonate	2016	9	2652
Failure to thrive [154]	Infant	2017	2	614
Neonatal seizures [155]	Neonate	2019	4	407
Neonatal encephalopathy [156]	Neonate	2017	8	286
Neonatal death [157]	Neonate	2016	16	222*
Neurodevelopmental delay [158]	Infant	2019	2	61
Low birthweight/small for gestational age	Neonate	NA#	1	5
Congenital microcephaly [159]	Neonate	2017	0	0

*Infant deaths may also be included in the 222.

# Date of publication has not been provided as these are composite reported study outcomes combining two GAIA outcomes (antepartum and postpartum genital bleeding, abortion & stillbirth, low birthweight and small for gestational age).

Retrospective cohort studies made up the majority in terms of study design (50.9%), twenty-four reported on randomised controlled trials (20.7%) and 23 (19.8%) were prospective cohort studies. Forty-eight studies (41.4 %) utilised electronic health records to identify AEFI. Most of the included studies focused on adverse events of special interest in pregnancy alone (65.5%), 32 (27.6%) assessed vaccine reactogenicity additionally and 6.9% focused solely on reactogenicity. Table two details these and other key characteristics of each study.

Most studies were conducted in high-income settings (78.4%). Of note, only 12 (10.3%) studies were exclusively conducted in lower- or lower-middle-income countries. Studies conducted in high-income settings tended to be larger than those in lower-income settings with a mean number of participants ten times higher than in low to upper-middle-income settings (11,162 v 996). No EMR or registry-based studies conducted in LMIC settings were identified.

### Reported adverse event outcomes

Table 3 outlines the frequency that AESIs defined by the GAIA-group were reported in the included studies. Hypertensive disorders of pregnancy (19,430 events) and chorioamnionitis (18,273) were the most frequently reported events in maternal subjects. Small for gestational age (30,929), preterm birth (28,828) and congenital anomalies (13,466) were the most frequent neonatal outcomes. There were many reported adverse events that have not been systematically defined, these include neonatal jaundice (17,166), macrosomia (2020), autism spectrum disorders and attention deficit hyperactivity disorders (1609) (Supplementary Table 6).

### Utilisation of brighton collaboration definitions

Eight of the included studies cited or used the Brighton Collaboration GAIA definitions in their publications (Table 4). Two studies cited the GAIA definitions in their discussion sections as a way of improving the quality of their respective studies but did not use the definitions [13], [14]]. Three studies [15], [16], [17] used the Brighton Collaboration’s 2015 publication [10] of key terms for the assessment of safety of vaccines in pregnancy to guide the outcomes they investigated and reported in their studies but did not use the published definitions; two of these were EHR-based and used ICD codes to identify cases [15], [16], one was a randomised controlled trial [17]. Three studies used the definition or a modified version of it. One of these used the GAIA stillbirth definition to differentiate between antepartum and intrapartum stillbirth but used other criteria for gestational age assessment [18]. A further study reported on several GAIA outcomes but modified some of the criteria, for example, including multi-organ complications (including small for gestational age) as one of the criteria for diagnosing pre-eclampsia [19]. Only one published study provided a level of certainty associated with the reported outcomes [20].

**Table 4:** Publications that cited or utilised GAIA Definitions.

Author & Citation	Vaccine	Definition Used	Modified?	Level of confidence?
Munoz et al, 2019 [17]	RSV	No	NA	NA
Panagiotakopoulos et al, 2020 [18]	Influenza & TDaP	Stillbirth	Yes	No
Mohammed et al, 2021 [19]	TDaP	Hypertensive disorders	Yes	No
Kharbanda et al, 2017 [20]	Influenza	Birth Defects	No	Yes
Skipetrova et al, 2018 [14]	Dengue	No	NA	NA
Petousis-Harris et al, 2019 [15]	TDaP	No	NA	NA
Griffin et al, 2018 [16]	TDaP	No	NA	NA
Calvert et al, 2022 [13]	SARS-CoV-2	No	NA	NA

### Thresholds and cut-offs

Criteria for many of the defined events depend on temporal or physiological thresholds for determining the presence of an event. These include, for example, the size of measured induration around a vaccine site reaction or the gestational age at which a fetal loss is classified as a stillbirth as opposed to a spontaneous abortion. Critically, several of the GAIA outcomes depend on accurate assessment of the gestational age of the pregnancy. The method of gestational age assessment was infrequently reported, even in randomised controlled trials where 55% did not provide the assessment methodology in their publication (or accompanying protocol where available).

Of the 39 studies that reported stillbirths, 12 different thresholds were published, these varied in terms of gestational age and/or weight thresholds, whilst 12 studies (30.8%) did not provide a definition at all (Supplementary Table 7). Similarly for preterm birth, 10 publications (14.7 %) did not provide a definition of the gestational age below which the birth was considered preterm (Supplementary Table 8).

### Serious adverse event reporting

Twenty-four publications reported on 22 different RCTs (Supplementary Table 9). Nineteen of these reported on serious adverse events in their published manuscripts. Eight studies (42.1%) provided a definition of an SAE in the manuscript or accompanying published protocol or provided a reference to the different standards they used for categorising SAEs. There were variations in the definition used; two defined an SAE as an event that was considered life threatening, prolonged hospitalisation, resulted in persistent or significant disability, or resulted in death [21], [22]. Four studies maintained that definition with the additional inclusion of congenital anomalies or birth defects [17], [23], [24], [25]. Additionally, two studies included all outcomes of special interest in pregnancy [26] as serious adverse events or a selection (spontaneous abortions, fetal death, stillbirth) [27] of adverse outcomes of special interest as SAEs.

## Discussion

This review has demonstrated that almost eight in ten comparative vaccine safety studies published between 2014 and the end of 2022 were undertaken in high-income settings. Where studies were set in lower-income settings, they tended to be smaller, recruiting fewer participants and not utilising large health registries or electronic sources. There are several reasons why vaccine safety concerns may differ by setting; safety profiles may differ based on factors such as nutritional status, presence of coexisting infections such as HIV, genetic factors, and exposure patterns. By conducting maternal vaccine safety studies in low-resource settings, our understanding of the safety profile of vaccines in diverse populations can be enhanced, addressing specific safety concerns relevant to these settings, and ensuring that vaccine recommendations are inclusive of all populations and evidence-based. These benefits can only be realised by improving the equitable distribution of vaccine research globally and investing resources in conducting larger vaccine safety studies in LMICs.

Over half of the studies that reported comparative vaccine safety in pregnant women identified in this review utilised retrospective study designs. The GAIA definitions were designed to be used prospectively in clinical trial settings, and may not be applicable in studies that utilise retrospective case identification. A 2022 review demonstrated that large numbers of cases identified retrospectively, and which utilised routine care data were unclassifiable using the GAIA definitions [9]. Safety data in pregnant women relies heavily on post-marketing pharmacovigilance and retrospective datasets such as electronic health-records, and billing codes on administrative data are commonly used in these study designs. Case definitions that can be applied to retrospective datasets and from regular clinical data are also needed. The GAIA definitions might need to be adapted for use in this context.

Significant work to evaluate the GAIA definitions has been undertaken, however, a recent review identified that half had yet to undergo formal evaluation in published studies [9]. These include some of the most frequently reported maternal (chorioamnionitis and post-partum haemorrhage), fetal (spontaneous abortions) and neonatal (congenital anomalies) AESIs identified in our systematic review. Work to complete validation of the definitions is planned and these commonly reported outcomes should be prioritised in future validation studies. Several recent publications have added to the weight of evidence in this area. Two studies from Democratic Republic of Congo have assessed the definitions in retrospective [28] and prospective [29] datasets and will help progress the validation process.

Our review also identified some commonly reported AESI that have not been systematically defined by the Brighton Collaboration, these include neonatal jaundice, macrosomia, autism spectrum disorder and attention deficit hyperactivity disorder. The comprehensive list of all reported AESI from maternal vaccine studies generated by this review could support identification of other outcomes beyond the 26 already published that require attention.

In agreement with the findings of the 2015 systematic review; there were substantial variations in temporal and physiological thresholds for reporting of adverse event outcomes, particularly around gestational age. Given that gestational age assessment is critical in maternal vaccine studies, not only for assessment of outcomes such as stillbirth, preterm birth and small for gestational age, but also for the actual timing of vaccine delivery, significant improvements could be made in reporting of the methodologies used in maternal vaccine studies. Our review identified that 14.7% of studies did not provide this information on the gestational age threshold for preterm birth and 55% of RCTs did not provide information about how the pregancy was dated in their published manuscripts.

This information is required so that a transparent assessment of the level of confidence associated with reported maternal outcomes such as hypertensive disorders, fetal outcomes such as fetal growth restriction and spontaneous abortion and neonatal outcomes such as small for gestational age can be made.

A 2012 systematic review of safety reporting in developing country vaccine clinical trials identified 50 RCTs between 1989 and 2011. AEFI definitions were used in 35 of 50 vaccine trials. Standardised Brighton Collaboration definitions were used in two trials. Logistic regression revealed a positive association between use of a fever case definition and the reporting rate for fever as an AEFI (p = 0.027) suggesting that use of a definition may increase the likelihood that the outcome was reported [30].

Seventy-nine studies (68.1 %) were published after the first set of GAIA definitions in 2016, but despite this, we have demonstrated poor uptake of the definitions even in the prospective RCTs for which they were developed. Similarly poor uptake of Brighton Collaboration definitions was identified in a 2019 study in low- and middle-income settings. The authors looked at all Brighton Collaboration definitions, not just those specifically relating to pregnancy and concluded that the Brighton Collaboration case definitions had not been broadly used or assessed for performance in low- and middle-income countries (LMICs). They recommended involving more LMIC scientists in the case definition working-groups, disseminating free webinars, publicly available recordings, and case definition documents. They also suggested formally assessing barriers for use in LMICs; and developing tools to support implementation [31]. Our review suggests that the problem is not limited to LMICs, and such activities should cover a wider range of settings.

Completion of the planned evaluation studies and undertaking critical revisions that have been identified for poorly performing definitions, such as stillbirth, could also improve confidence in the definitions and support more widespread adoption [32]. Given the importance of consistency of safety reporting, consideration should also be given to making use of the definitions by investigators a requirement. The CONSORT (Consolidated Standards of Reporting Trials) statement; a structured framework for researchers to report their study methods and findings transparently and accurately, helps improve the quality and reliability of published research [33]. Whilst use of the framework isn’t mandatory, many medical journals and publishers strongly encourage or require authors to follow the CONSORT guidelines when submitting RCTs for publication. Moreover, many research funding agencies and ethics committees also recommend or require compliance with the CONSORT guidelines when researchers apply for grants or ethical approvals. Given the importance of robust vaccine safety monitoring, and the limited uptake of the definitions, a similar approach should be considered for standardised reporting of adverse events of special interest in maternal vaccine trials once the evaluation and revision process has been completed.

Our search was undertaken in four major literature databases, but we did not hand-search individual journals or grey literature which may have led to us missing a small number of relevant papers. In addition, it was not feasible to assess vaccine studies that did not include a comparator group, studies undertaken using passive surveillance registries, such as VAERS, were therefore excluded. Studies were limited to those published in English and so relevant studies from LMIC settings written in other languages may have been missed. We think that this is likely to have a limited impact on the overall number of comparative safety studies identified from LMICs. Fulton et al did not limit their database search to English and only identified 5/5488 (0.09 %) potentially relevant non-English language studies [8]. It was also beyond the scope of our study to contact the authors of each study to request additional, unpublished information regarding AEFI definitions, we did however, review any protocols accompanied alongside the main manuscript.

## Conclusions

Research into vaccine safety continues to be undertaken predominantly in high-income regions, while low-income countries receive insufficient attention, both in terms of the quantity of studies conducted and the number of participants involved. Vaccines against diseases such as Ebola and malaria, that may be of particular benefit to pregnant women, are being developed for, and introduced in, high-burden LMIC settings. To ensure that the introduction of these vaccines is supported by adequate safety data, it is essential to prioritise the establishment of capacity and infrastructure for conducting rigorous studies in these settings.

There remains a persistent issue of inconsistency, variability, and a lack of transparency in reporting adverse events in maternal vaccine studies. Furthermore, the adoption of GAIA (Global Alignment of Immunization Safety Assessment in pregnancy) AESI case definitions, since their publication, has been disappointingly low, with only one study providing a level of confidence associated with their reported outcomes. However, it is important to note that even though the majority of studies were published after the definitions, a large number of the studies included in this review began recruitment before their publication, and it may take some time for widespread adoption to occur.

To address this, there is a need to prioritise the evaluation of the thirteen GAIA definitions that have not yet been tested in published studies. This step will ensure that these definitions have undergone thorough assessment, instilling confidence in researchers to use them. Additionally, the planned review and revision of the case definitions may contribute to greater adoption. Finally, consideration should be given to ways in which the definitions and associated materials can be disseminated more widely within the research community.

## Disclaimer

The authors have no interests to declare. P Lambach works for the World Health Organization (WHO). The authors alone are responsible for the views expressed in this publication and they do not necessarily represent the decisions, policy or views of the WHO.

## CRediT authorship contribution statement

**Hannah Davies:** . **Emma Thorley:** Data curation, Project administration, Writing – review & editing. **Rossul Al-Bahadili:** Data curation, Writing – review & editing. **Natalina Sutton:** Data curation, Writing – review & editing. **Jessica Burt:** Data curation, Writing – review & editing. **Lauren Hookham:** Data curation, Writing – review & editing. **Kostas Karampatsas:** Data curation, Writing – review & editing. **Philipp Lambach:** Conceptualization, Writing – review & editing. **Flor Munoz:** Writing – review & editing. **Clare Cutland:** Conceptualization, Writing – review & editing. **Saad Omer:** Conceptualization, Data curation, Writing – review & editing. **Kirsty Le Doare:** Conceptualization, Data curation, Writing – review & editing.

## Declaration of competing interest

Financial support for this work was provided by the European and Developing Countries Clinical Trials Partnership. Clare Cutland and Flor Muñoz were involved in leading the GAIA definition development. Hannah Davies is involved in the GAIA definition revision process.

## Data Availability

Data will be made available on request.
