# Corrigendum to “Defining and reporting adverse events of special interest in comparative maternal vaccine studies: a systematic review”. [Vaccine X, 2024 Feb 23;18:100464]

**DOI:** 10.1016/j.jvacx.2025.100612

**Published:** 2025-02-05

**Authors:** Hannah G. Davies, Emma V. Thorley, Rossul Al-Bahadili, Natalina Sutton, Jessica Burt, Lauren Hookham, Kostas Karampatsas, Philipp Lambach, Flor Muñoz, Clare L. Cutland, Saad Omer, Kirsty Le Doare

**Affiliations:** a Centre for Neonatal and Paediatric Infection, Institute of Infection & Immunity, St George's, University of London, Cranmer Terrace, Tooting, London, United Kingdom; b MRC, UVRI & LSHTM Uganda Research Centre, Entebbe, Uganda; c Makerere University John Hopkins Research Unit, Kampala, Uganda; d World Health Organization, Geneva, Switzerland; e Paediatric Infectious Diseases Department, Baylor College of Medicine, Houston, TX, USA; f Wits African Leadership in Vaccinology Expertise (Wits-Alive), School of Pathology, Faculty of Health Science, University of the Witwatersrand, Johannesburg, South Africa; g O'Donnell School of Public Health, UT Southwestern Medical Center, Texas, USA

The funding source was not appropriately acknowledged in the original article. The funding statement should have read as follows:

“This project is part of the EDCTP2 programme (grant number RIA2018V-2304-PREPARE) supported by the 10.13039/501100000780European Union.”

