# Supplementary material for: Defining and reporting adverse events of special interest in comparative maternal vaccine studies: a systematic review
Source: Vaccine X. 2024 Feb 23;18:100464. doi: 10.1016/j.jvacx.2024.100464 (PMC10943481; doi:10.1016/j.jvacx.2024.100464)
Supplement: Supplementary data 1 [file mmc1.docx]

Supplementary Table 1 - Medline Search Strategy

|  | Search Term | Hits |
| --- | --- | --- |
|  | MESH Vaccines | 36456 |
|  | MESH Vaccination | 135110 |
|  | MESH Immunization | 61354 |
|  | Vaccin* OR immunis* OR immuniz* OR inoculat* | 273669 |
|  | 1 OR 2 OR 3 OR 4 | 1168563 |
|  | MESH Pregnancy | 267163 |
|  | MESH Mothers | 76795 |
|  | MESH Prenatal care | 42632 |
|  | MESH Maternal exposure | 16448 |
|  | Matern* OR Mother* OR prenat* OR antenat* Pregnan* | 1719446 |
|  | 6 OR 7 OR 8 OR 9 OR 10 | 396411 |
|  | MESH "Drug-Related Side Effects and Adverse Reactions"/ | 49711 |
|  | MESH Safety/ | 50054 |
|  | MESH Risk Factors/ | 1304515 |
|  | MESH Risk Management/ | 23524 |
|  | (Adverse OR injurious OR undesirable) adj3 (event* our outcome* or effect* or reaction*) | 3115742 |
|  | Risk* OR safety OR side effect* or aefi* | 5640433 |
|  | 12 OR 13 OR 14 OR 15 OR 16 OR 17 | 2408271 |
|  | 5 AND 11 AND 18 | 5764 |
|  | Line 19 Limited to (English language and humans) | 4516 |
|  | **Line 20 limited to date of the previous search** | **3998** |

Supplementary Table 2 - Embase Search Strategy

|  | Search Term | Hits |
| --- | --- | --- |
|  | MESH Vaccine | 103190 |
|  | MESH Vaccination | 285205 |
|  | MESH Immunisation | 143065 |
|  | Vaccin* OR immunis* OR immuniz* OR inoculat* | 1144390 |
|  | 1 or 2 or 3 or 4 | 1144390 |
|  | MESH Pregnancy | 757689 |
|  | MESH Mother | 149742 |
|  | MESH Prenatal care | 69981 |
|  | MESH Maternal exposure | 7686 |
|  | Matern* OR Mother* OR Pregnan* OR antenat* OR prenat* | 2097011 |
|  | 6 or 7 or 8 or 9 or 10 | 2097011 |
|  | MESH "side effect”/ | 526369 |
|  | MESH adverse event | 142083 |
|  | MESH adverse drug reaction | 389103 |
|  | MESH Safety/ | 353261 |
|  | MESH Risk Factor/ | 1913054 |
|  | MESH Risk Management/ | 62230 |
|  | (Adverse OR injurious or undesirable) adj3 (event our outcome* or effect* or reaction*) | 3053060 |
|  | Risk* OR safety OR side effect* or aefi* | 9733320 |
|  | 12 or 13 or 14 or 15 or 16 or 17 or 18 or 19 | 10945476 |
|  | 5 and 11 and 20 | 27027 |
|  | English language and humans | 23136 |
|  | **Line 22 limited to the date of the previous search** | **8369** |

Supplementary Table 3 - Cochrane Search Strategy

|  | Search Term | Hits |
| --- | --- | --- |
|  | MESH Vaccines | 32635 |
|  | MESH Immunization | 15822 |
|  | MESH Vaccination | 8369 |
|  | Vaccin* OR immunis* OR immuniz* OR inoculat* | 96769 |
|  | 1 OR 2 OR 3 OR 4 | 97176 |
|  | MESH Pregnancy | 72226 |
|  | MESH Mothers | 6357 |
|  | MESH Prenatal care | 4915 |
|  | MESH Maternal exposure | 198 |
|  | Matern* OR Mother* OR Pregnan* OR antenat* OR prenat* | 286709 |
|  | 6 OR 7 OR 8 OR 9 OR 10 | 287319 |
|  | MESH "Drug-Related Side Effects and Adverse Reactions"/ | 9348 |
|  | MESH Safety/ | 11463 |
|  | MESH Risk Factors/ | 77542 |
|  | MESH Risk Management/ | 19582 |
|  | Adverse OR injurious OR undesirable NEAR event* our outcome* or effect* or reaction* | 4046449 |
|  | Risk* OR safety OR side effect* or aefi* | 1677723 |
|  | 12 OR 13 OR 14 OR 15 OR 16 OR 17 | 4344260 |
|  | 5 AND 11 AND 17 | 8920 |
|  | **Line 19 limited to 2014-2021** | **2685** |

Supplementary Table 4 - Web of Science Search Strategy

|  | Search Term | Hits |
| --- | --- | --- |
| 1 | Vaccin* or immunis* or immuniz* or incoulat* | 300938 |
| 2 | Pregnan* or mother* or matern* or antenat* or prenat* | 462808 |
| 3 | Safety or risk* or side effect* or aefi* | 2973412 |
| 4 | Adverse or injurious or undesirable NEAR/3 event* or outcome* or effect* or reaction* | 8772639 |
| 5. | 3 or 4 | 10179499 |
| 6 | **Lines 1 AND 2 AND 5** | **9698** |

Supplementary Figure 1 - PRISMA Flow-chart

Records removed *before screening*:

Duplicate records removed **(n=10013)**

Records marked as ineligible by automation tools (n=0)

Records screened

**(n =14737)**

Records excluded**

**(n =14302)**

Records sought for retrieval

(**n=435)**

Records not retrieved **(n=3)**

Records assessed for eligibility

**(n=432)**

Reports excluded**: (n=256)**

Original research studies included in review

**(n =116)**

Reviews (**n=60)**

**Identification of studies via databases and registers**

**Identification**

**Screening**

**Included**

Records identified from:

Databases **(n=24750)**

Reason 1 = Pregnant women not the target population

Reason 2= Paper not in English/translatable

Reason 3= No analysis of vaccine safety as primary or secondary outcome

Reason 4 = Article is a letter, discussion, review, protocol or conference abstract

Reason 5 = No control group included

Reason 6 = Included in the previous review

Reason 7 = Another paper reporting results from study included

Reason 8 = Other

Supplementary Table 5 - Table of included review papers.

| **Number** | **Manuscript Title** | **Vaccine(s)** |
| --- | --- | --- |
|  | Impact of Haemophilus influenzae type B (Hib) and viral influenza vaccinations in pregnancy for improving maternal, neonatal and infant health outcomes.^1^ | Haemophilus |
|  | Vaccines for women for preventing neonatal tetanus.^2^ | Tetanus |
|  | Pneumococcal vaccination during pregnancy for preventing infant infection.^3^ | Pneumococcus |
|  | Prophylactic vaccination against human papillomaviruses to prevent cervical cancer and its precursors.^4^ | Human Papillomavirus |
|  | Vaccines for preventing influenza in healthy adults. ^5^ | Influenza |
|  | Systematic review of the clinical development of group B streptococcus serotype-specific capsular polysaccharide-based vaccines. ^6^ | Group B Streptococcus |
|  | A comprehensive review of influenza and influenza vaccination during pregnancy. ^7^ | Influenza |
|  | Safety profile of rubella vaccine administered to pregnant women: A systematic review of pregnancy related adverse events following immunisation, including congenital rubella syndrome and congenital rubella infection in the foetus or infant. ^8^ | Rubella |
|  | Safety of immunization during pregnancy: a review of the evidence of selected inactivated and live attenuated vaccines. ^9^ | Multiple |
|  | Safety, immunogenicity and risk-benefit analysis of rVSV-DELTAG-ZEBOV-GP (V920) Ebola vaccine in Phase I-III clinical trials across regions.^10^ | Ebola |
|  | Real-world evidence of quadrivalent meningococcal conjugate vaccine safety in the United States: a systematic review. ^11^ | Meningococcus |
|  | Safety of Tetanus, Diphtheria, and Pertussis Vaccination During Pregnancy: A Systematic Review.^12^ | Tetanus, Diphtheria, and Pertussis |
|  | Should expectant mothers be vaccinated against flu? A safety review.^13^ | Influenza |
|  | Peri-conceptional or pregnancy exposure of HPV vaccination and the risk of spontaneous abortion: a systematic review and meta-analysis. ^14^ | Human Papillomavirus |
|  | Fetal death and preterm birth associated with maternal influenza vaccination: systematic review. ^15^ | Influenza |
|  | A systematic review and meta-analysis of fetal outcomes following the administration of influenza A/H1N1 vaccination during pregnancy. ^16^ | Influenza |
|  | Immunogenicity, duration of protection, effectiveness and safety of rubella containing vaccines: A systematic literature review and meta-analysis.^17^ | Rubella |
|  | Varicella virus vaccine live: A 22-year review of postmarketing safety data. ^18^ | Varicella |
|  | Influenza vaccination during pregnancy: a systematic review of fetal death, spontaneous abortion, and congenital malformation safety outcomes. ^19^ | Influenza |
|  | Maternal vaccination against pertussis: a systematic review of the recent literature. ^20^ | Pertussis |
|  | Final report on exposure during pregnancy from a pregnancy registry for quadrivalent human papillomavirus vaccine. ^21^ | Human Papillomavirus |
|  | Maternal Influenza Vaccination and Risk for Congenital Malformations: A Systematic Review and Meta-analysis. ^22^ | Influenza |
|  | Safety of oral cholera vaccines during pregnancy in developing countries. ^23^ | Cholera |
|  | Review on the effects of influenza vaccination during pregnancy on preterm births. ^24^ | Influenza |
|  | An Overview of Quadrivalent Human Papillomavirus Vaccine Safety: 2006 to 2015. ^25^ | Human Papillomavirus |
|  | Safety of Maternal Immunization Against Pertussis: A Systematic Review. ^26^ | Pertussis |
|  | The safety of inactivated influenza vaccines in pregnancy for birth outcomes: a systematic review. ^27^ | Influenza |
|  | Literature review of vaccine-related adverse events reported from HPV vaccination in randomized controlled trials. ^28^ | Human Papillomavirus |
|  | Pregnancy outcomes after a mass vaccination campaign with an oral cholera vaccine: a systematic review and meta-analysis. ^29^ | Cholera |
|  | Pregnancy Outcomes After Human Papillomavirus Vaccination in Periconceptional Period or During Pregnancy: A Systematic Review and Meta-analysis. ^30^ | Human Papillomavirus |
|  | Safety and effectiveness of acellular pertussis vaccination during pregnancy: a systematic review. ^31^ | Pertussis |
|  | Efficacy and safety of pertussis vaccination for pregnant women - a systematic review of randomised controlled trials and observational studies. ^32^ | Pertussis |
|  | Risks Associated With Smallpox Vaccination in Pregnancy: A Systematic Review and Meta-analysis. ^33^ | Smallpox |
|  | The Effects of Influenza Vaccination during Pregnancy on Birth Outcomes: A Systematic Review and Meta-Analysis.^34^ | Influenza |
|  | Safety of administering live vaccines during pregnancy: A systematic review and meta-analysis of pregnancy outcomes. ^35^ | Mixed |
|  | Effects of maternal influenza vaccination on adverse birth outcomes: A systematic review and Bayesian meta-analysis. ^36^ | Influenza |
|  | Is HPV vaccination in pregnancy safe? ^37^ | Human Papillomavirus |
|  | Maternal influenza immunization and birth outcomes of stillbirth and spontaneous abortion: a systematic review and meta-analysis. ^38^ | Influenza |
|  | Yellow fever vaccine and risk of developing serious adverse events: a systematic review. ^39^ | Yellow fever |
|  | Safety and adverse events of prophylactic HPV vaccines among healthy women: A systematic review & meta analysis. ^40^ | Human Papillomavirus |
|  | Rabies post-exposure prophylaxis: A systematic review on abridged vaccination schedules and the effect of changing administration routes during a single course. ^41^ | Rabies |
|  | Adverse events associated with the use of recommended vaccines during pregnancy: An overview of systematic reviews ^42^ | Mixed |
|  | One "misunderstood" health issue: demonstrating and communicating the safety of influenza a vaccination in pregnancy: a systematic review and meta-analysis. ^43^ | Influenza |
|  | Quadrivalent HPV vaccine safety review and safety monitoring plans for nine-valent HPV vaccine in the United States. ^44^ | Human Papillomavirus |
|  | Efficacy and safety of prophylactic HPV vaccines. A Cochrane review of randomized trials. ^45^ | Human Papillomavirus |
|  | Influenza vaccination during pregnancy: A systematic review of effectiveness and safety. ^46^ | Influenza |
|  | Systematic review and meta-analysis of the effect of pertussis vaccine in pregnancy on the risk of chorioamnionitis, non-pertussis infectious diseases and other adverse pregnancy outcomes. ^47^ | Pertussis |
|  | Vaccines for preventing influenza in healthy children. ^48^ | Influenza |
|  | Maternal influenza and pregnancy outcomes-a systematic review of comparative studies^49^ | Influenza |
|  | Immunity after COVID-19 vaccination in people with higher risk of compromised immune status: a scoping review. ^50^ | SARS-CoV-2 |
|  | Maternal vaccination-current status, challenges, and opportunities. ^51^ | Mixed |
|  | Effectiveness and safety of COVID-19 vaccine in pregnant women: a systematic review with meta-analysis. ^52^ | SARS-CoV-2 |
|  | Complications of COVID-19 Vaccines during Pregnancy; a Systematic Review. ^53^ | SARS-CoV-2 |
|  | Is it safe and effective to administer COVID-19 vaccines during pregnancy? A systematic review and meta-analysis. ^54^ | SARS-CoV-2 |
|  | The consequences of COVID-19 and its vaccine on pregnant and lactating mothers. ^55^ | SARS-CoV-2 |
|  | COVID-19 vaccination in pregnancy. ^56^ | SARS-CoV-2 |
|  | COVID-19 Vaccination and Pregnancy. ^57^ | SARS-CoV-2 |
|  | COVID-19 vaccines in pregnancy. ^58^ | SARS-CoV-2 |
|  | Peripartum Outcomes Associated With COVID-19 Vaccination During Pregnancy A Systematic Review and Meta-analysis. ^59^ | SARS-CoV-2 |
|  | The impact of maternal SARS-CoV-2 infection and COVID-19 vaccination on maternal-fetal outcomes. ^60^ | SARS-CoV-2 |

Supplementary Table 6 - Reported events specific to pregnancy or infancy with no standardised GAIA definition.

| Outcome | Studies reporting cases | Number of reported cases in vaccinees |
| --- | --- | --- |
| Caesarean or instrumental delivery | 8 | 50713 |
| Hospital admissions | 20 | 39608 |
| Neonatal jaundice | 2 | 17166 |
| Low APGAR scores | 16 | 6534 |
| Serious adverse event/adverse event/adverse event of special interest (totals) | 16 | 2747 |
| Macrosomic baby | 3 | 2020 |
| Autism/Autism Spectrum Disorder or  Attention Deficit Hyperactivity Disorder | 4 | 1609 |
| Transient tachypnoea of the newborn | 2 | 974 |
| Prolonged or pre-labour rupture of membranes | 4 | 678 |
| Induction of labour | 4 | 676 |
| Mechanical ventilation | 5 | 573 |
| Peripartum fever | 4 | 525 |
| Anaemia | 3 | 287 |
| Pulmonary hypertension | 1 | 250 |
| Infections | 12 | 181 |
| Term delivery | 1 | 109 |
| Termination of pregnancy | 7 | 83 |
| Neonatal resuscitation | 1 | 58 |
| Lactation disorder | 1 | 55 |
| Perinatal death | 3 | 32 |
| Thrombocytopaenia or HELLP | 6 | 27 |
| Meconium aspiration | 3 | 15 |
| Oligohydramnios | 5 | 5 |
| Malpresentation | 3 | 2 |
| Other outcome | 26 | 218 |
| Total |  | **125145** |

Supplementary Table 7 – Gestational-age thresholds for stillbirth in studies that reported cases in the investigational or vaccinee groups.

| Stillbirths (n=39) | Number (%) |
| --- | --- |
| ≥ 20 weeks’ gestation | 9 (23.1) |
| ≥ 20 weeks’ gestation or ≥ 400g | 1 (2.6) |
| > 20 weeks’ gestation | 2 (5.1) |
| ≥ 21 weeks’ gestation | 1 (5.1) |
| ≥ 22 weeks’ gestation | 4 (10.3) |
| > 22 weeks’ gestation | 1 (2.6) |
| ≥ 23 weeks’ gestation | 1 (2.6) |
| > 23 weeks’ gestation | 1 (2.6) |
| ≥ 24 weeks gestation | 1 (2.6) |
| > 24 weeks gestation | 1 (2.6) |
| 180 days amenorrhoea | 1 (2.6) |
| ≥ 28 weeks’ gestation | 2 (5.1) |
| >28 weeks’ gestation | 1 (2.6) |
| Brighton Collaboration definition used | 1 (2.6) |
| No gestational age threshold published | 12 (30.8) |
| Total | **39 (100)** |

Supplementary Table 8 - Gestational-age thresholds for preterm birth in studies that reported cases in the interventional group or vaccinees.

| Preterm birth (n=68) | Number (%) |
| --- | --- |
| <37 weeks’ gestation | 50 (73.5) |
| < 35 weeks’ gestation | 1 (1.5) |
| < 36 weeks’ gestation | 1 (1.5) |
| >20 weeks and <37 weeks’ gestation | 2 (2.9) |
| >20 weeks, >400g and <37 weeks’ gestation | 1 (1.5) |
| ≥22 weeks to 37 weeks’ gestation | 1 (1.5) |
| ≥24 weeks to 37 weeks’ gestation | 1 (1.5) |
| <37+6 weeks | 1 (1.5) |
| No specific threshold defined in the publication | 10 (14.7) |
| Total | **68** |

Supplementary Table 9 - Key information about the randomised controlled trials included in the review.

| Author | Year of publication | Country | | Vaccine | Brand and/or formulation |
| --- | --- | --- | --- | --- | --- |
| *Binks et al^61^  McHugh et al^62^ | 2015  2020 | | Australia | Pneumococcal | 23vPPV ﻿PNEUMOVAX® 23 |
| Barug et al^63^ | 2019 | | Netherlands | TDaP | ﻿Boostrix |
| Tapia et al^64^ | 2016 | | Mali | Influenza | ﻿Vaxigrip |
| Madhi et al^65^ | 2020 | | Argentina, Australia, Chile, Bangladesh, South Africa, The Philippines, Mexico, Spain, UK, USA | RSV | ﻿RSV F vaccine |
| Donders et al^66^ | 2016 | | Belgium, Canada | GBS | Trivalent CRM﻿197- conjugated GBS vaccine |
| Munoz et al^67^ | 2019 | | USA | RSV | RSV F |
| *Steinhoff et al^68^  Kozuki^69^ | 2017 | | Nepal | Influenza | ﻿Vaxigrip |
| Nunes et al^70^ | 2020 | | South Africa | Influenza | ﻿Vaxigrip |
| Madhi et al^71^ | 2016 | | South Africa | GBS | ﻿Non-adjuvanted trivalent GBS vaccine |
| Perrett et al^72^ | 2020 | | Australia, Canada, Czech Repblic, Finland, Italy, Spain | TDaP | Boostrix |
| Swamy et al^73^ | 2020 | | USA | GBS | Trivalent |
| Munoz et al^74^ | 2020 | | USA | Influenza | IIV3 (Agriflu/Fluzone/Fluarix), ﻿Sanofi Pasteur Inc, ﻿GlaxoSmithKline Biologicals, |
| Hoang et al^75^ | 2016 | | Vietnam | TDaP | ﻿Adacel® |
| Kotinov et al^76^ | 2018 | | Russia | Influenza | Agrippal, Grippol-Plus |
| ﻿Wanlapakorn et al^77^ | 2018 | | Thailand | TDaP | Boostrix |
| Vesikari et al^78^ | 2020 | | Finland | Influenza | IIV4 |
| ﻿Simões et al^79^ | 2019 | | South Africa | Influenza | IIV3 ﻿Vaxigrip |
| Skipetrova et al^80^ | 2018 | | Mixed | Dengue | ﻿CYD-TDV |
| Villareal Perez^81^ | 2017 | | Mexico | TDaP |  |
| Halperin et al^82^ | 2018 | | Canada | Tdap | Not specified |
| Munoz et al^83^ | 2018 | | USA | Influenza & H1N1 | Fluzone or Fluarix |
| Weinberg et al^84^ | 2021 | | Brazil | Pneumococcal | PCV10 & PCV23 |

*Two studies included reporting on one trial – considered together for description of safety reporting.

1. Salam RA, Das JK, Dojo Soeandy C, Lassi ZS, Bhutta ZA. Impact of Haemophilus influenzae type B (Hib) and viral influenza vaccinations in pregnancy for improving maternal, neonatal and infant health outcomes. Cochrane Database Syst Rev [Internet]. 2015;(6):CD009982. Available from: http://ovidsp.ovid.com/ovidweb.cgi?T=JS&PAGE=reference&D=med12&NEWS=N&AN=26059051

2. Demicheli V, Barale A, Rivetti A. Vaccines for women for preventing neonatal tetanus. Cochrane Database Syst Rev [Internet]. 2015;(7):CD002959. Available from: http://ovidsp.ovid.com/ovidweb.cgi?T=JS&PAGE=reference&D=med12&NEWS=N&AN=26144877

3. Chaithongwongwatthana S, Yamasmit W, Limpongsanurak S, Lumbiganon P, Tolosa JE. Pneumococcal vaccination during pregnancy for preventing infant infection. Cochrane Database Syst Rev [Internet]. 2015;1:CD004903. Available from: http://ovidsp.ovid.com/ovidweb.cgi?T=JS&PAGE=reference&D=med12&NEWS=N&AN=25613573

4. Arbyn M, Xu L, Simoens C, Martin-Hirsch PP. Prophylactic vaccination against human papillomaviruses to prevent cervical cancer and its precursors. Cochrane Database Syst Rev [Internet]. 2018;5:CD009069. Available from: http://ovidsp.ovid.com/ovidweb.cgi?T=JS&PAGE=reference&D=med15&NEWS=N&AN=29740819

5. Demicheli V, Jefferson T, Ferroni E, Rivetti A, Di Pietrantonj C. Vaccines for preventing influenza in healthy adults. Cochrane Database Syst Rev [Internet]. 2018;2:CD001269. Available from: http://ovidsp.ovid.com/ovidweb.cgi?T=JS&PAGE=reference&D=med15&NEWS=N&AN=29388196

6. Dzanibe S, Madhi SA. Systematic review of the clinical development of group B streptococcus serotype-specific capsular polysaccharide-based vaccines. Expert Rev Vaccines [Internet]. 2018;17(7):635–51. Available from: http://ovidsp.ovid.com/ovidweb.cgi?T=JS&PAGE=reference&D=med15&NEWS=N&AN=29961350

7. Yuen CYS, Tarrant M. A comprehensive review of influenza and influenza vaccination during pregnancy. J Perinat Neonatal Nurs [Internet]. 2014;28(4):261–70. Available from: http://ovidsp.ovid.com/ovidweb.cgi?T=JS&PAGE=reference&D=med11&NEWS=N&AN=25347105

8. Mangtani P, Evans SJW, Lange B, Oberle D, Smith J, Drechsel-Baeuerle U, et al. Safety profile of rubella vaccine administered to pregnant women: A systematic review of pregnancy related adverse events following immunisation, including congenital rubella syndrome and congenital rubella infection in the foetus or infant. Vaccine [Internet]. 2020;38(5):963–78. Available from: http://ovidsp.ovid.com/ovidweb.cgi?T=JS&PAGE=reference&D=medl&NEWS=N&AN=31839467

9. Keller-Stanislawski B, Englund JA, Kang G, Mangtani P, Neuzil K, Nohynek H, et al. Safety of immunization during pregnancy: a review of the evidence of selected inactivated and live attenuated vaccines. Vaccine [Internet]. 2014;32(52):7057–64. Available from: http://ovidsp.ovid.com/ovidweb.cgi?T=JS&PAGE=reference&D=med11&NEWS=N&AN=25285883

10. Bache BE, Grobusch MP, Agnandji ST. Safety, immunogenicity and risk-benefit analysis of rVSV-DELTAG-ZEBOV-GP (V920) Ebola vaccine in Phase I-III clinical trials across regions. Future Microbiol [Internet]. 2020;15:85–106. Available from: http://ovidsp.ovid.com/ovidweb.cgi?T=JS&PAGE=reference&D=medl&NEWS=N&AN=32030996

11. Becerra-Culqui T a, Sy LS, Solano Z, Tseng HF. Real-world evidence of quadrivalent meningococcal conjugate vaccine safety in the United States: a systematic review. Hum Vaccin Immunother [Internet]. 2021;17(5):1432–41. Available from: http://www.tandfonline.com/loi/khvi20

12. McMillan M, Clarke M, Parrella A, Fell DB, Amirthalingam G, Marshall HS. Safety of Tetanus, Diphtheria, and Pertussis Vaccination During Pregnancy: A Systematic Review. Obstetrics and gynecology [Internet]. 2017;129(3):560–73. Available from: http://ovidsp.ovid.com/ovidweb.cgi?T=JS&PAGE=reference&D=med14&NEWS=N&AN=28178054

13. Loubet P, Kerneis S, Anselem O, Tsatsaris V, Goffinet F, Launay O. Should expectant mothers be vaccinated against flu? A safety review. Expert Opin Drug Saf [Internet]. 2014;13(12):1709–20. Available from: http://ovidsp.ovid.com/ovidweb.cgi?T=JS&PAGE=reference&D=med11&NEWS=N&AN=25363497

14. Tan J, Xiong YQ, He Q, Liu YM, Wang W, Chen M, et al. Peri-conceptional or pregnancy exposure of HPV vaccination and the risk of spontaneous abortion: a systematic review and meta-analysis. BMC Pregnancy Childbirth [Internet]. 2019;19(1):302. Available from: http://ovidsp.ovid.com/ovidweb.cgi?T=JS&PAGE=reference&D=med16&NEWS=N&AN=31426762

15. Fell DB, Platt RW, Lanes A, Wilson K, Kaufman JS, Basso O, et al. Fetal death and preterm birth associated with maternal influenza vaccination: systematic review. BJOG [Internet]. 2015;122(1):17–26. Available from: http://ovidsp.ovid.com/ovidweb.cgi?T=JS&PAGE=reference&D=med12&NEWS=N&AN=25040307

16. Zhang C, Wang X, Liu D, Zhang L, Sun X. A systematic review and meta-analysis of fetal outcomes following the administration of influenza A/H1N1 vaccination during pregnancy. Int J Gynaecol Obstet [Internet]. 2018;141(2):141–50. Available from: http://ovidsp.ovid.com/ovidweb.cgi?T=JS&PAGE=reference&D=med15&NEWS=N&AN=29149524

17. de Gier B, de Oliveira Bressane Lima P, de Melker HE, Hahne SJM, Veldhuijzen IK, van den Boogaard J, et al. Immunogenicity, duration of protection, effectiveness and safety of rubella containing vaccines: A systematic literature review and meta-analysis. Vaccine [Internet]. 2021;39(6):889–900. Available from: http://www.elsevier.com/locate/vaccine

18. Woodward M, Marko A, Galea S, Eagel B, Straus W. Varicella virus vaccine live: A 22-year review of postmarketing safety data. Open Forum Infect Dis [Internet]. 2019;6(8):ofz295. Available from: https://academic.oup.com/ofid

19. McMillan M, Porritt K, Kralik D, Costi L, Marshall H. Influenza vaccination during pregnancy: a systematic review of fetal death, spontaneous abortion, and congenital malformation safety outcomes. Vaccine [Internet]. 2015;33(18):2108–17. Available from: http://ovidsp.ovid.com/ovidweb.cgi?T=JS&PAGE=reference&D=med12&NEWS=N&AN=25758932

20. Gkentzi D, Katsakiori P, Marangos M, Hsia Y, Amirthalingam G, Heath PT, et al. Maternal vaccination against pertussis: a systematic review of the recent literature. Arch Dis Child Fetal Neonatal Ed [Internet]. 2017;102(5):F456–63. Available from: http://ovidsp.ovid.com/ovidweb.cgi?T=JS&PAGE=reference&D=med14&NEWS=N&AN=28468899

21. Goss MA, Lievano F, Buchanan KM, Seminack MM, Cunningham ML, Dana A. Final report on exposure during pregnancy from a pregnancy registry for quadrivalent human papillomavirus vaccine. Vaccine [Internet]. 2015;33(29):3422–8. Available from: http://ovidsp.ovid.com/ovidweb.cgi?T=JS&PAGE=reference&D=med12&NEWS=N&AN=25869893

22. Polyzos KA, Konstantelias AA, Pitsa CE, Falagas ME. Maternal Influenza Vaccination and Risk for Congenital Malformations: A Systematic Review and Meta-analysis. Obstetrics and gynecology [Internet]. 2015;126(5):1075–84. Available from: http://ovidsp.ovid.com/ovidweb.cgi?T=JS&PAGE=reference&D=med12&NEWS=N&AN=26444106

23. Khan AI, Islam MT, Qadri F. Safety of oral cholera vaccines during pregnancy in developing countries. Hum Vaccin Immunother [Internet]. 2017;13(10):2245–6. Available from: http://ovidsp.ovid.com/ovidweb.cgi?T=JS&PAGE=reference&D=med14&NEWS=N&AN=28825876

24. Nunes MC, Madhi SA. Review on the effects of influenza vaccination during pregnancy on preterm births. Hum Vaccin Immunother [Internet]. 2015;11(11):2538–48. Available from: http://ovidsp.ovid.com/ovidweb.cgi?T=JS&PAGE=reference&D=med12&NEWS=N&AN=26267701

25. Vichnin M, Bonanni P, Klein NP, Garland SM, Block SL, Kjaer SK, et al. An Overview of Quadrivalent Human Papillomavirus Vaccine Safety: 2006 to 2015. Pediatr Infect Dis J [Internet]. 2015;34(9):983–91. Available from: http://ovidsp.ovid.com/ovidweb.cgi?T=JS&PAGE=reference&D=med12&NEWS=N&AN=26107345

26. D’Heilly C, Macina D, Switzer C. Safety of Maternal Immunization Against Pertussis: A Systematic Review. Infect Dis Ther [Internet]. 2019;8(4):543–68. Available from: http://www.springer.com/springer+healthcare/journal/40121

27. Giles ML, Krishnaswamy S, Macartney K, Cheng A. The safety of inactivated influenza vaccines in pregnancy for birth outcomes: a systematic review. Hum Vaccin Immunother [Internet]. 2019;15(3):687–99. Available from: http://ovidsp.ovid.com/ovidweb.cgi?T=JS&PAGE=reference&D=med16&NEWS=N&AN=30380986

28. Macki M, Dabaja AA. Literature review of vaccine-related adverse events reported from HPV vaccination in randomized controlled trials. Basic Clin Androl. 2016;26.

29. Zhang Y, Zhang H, Wang B, Song G, Hayden JC, Amirthalingam P, et al. Pregnancy outcomes after a mass vaccination campaign with an oral cholera vaccine: a systematic review and meta-analysis. BJOG [Internet]. 2020;127(9):1066–73. Available from: http://ovidsp.ovid.com/ovidweb.cgi?T=JS&PAGE=reference&D=med17&NEWS=N&AN=32289871

30. Wang A, Liu C, Wang Y, Yin A, Wu J, Zhang C, et al. Pregnancy Outcomes After Human Papillomavirus Vaccination in Periconceptional Period or During Pregnancy: A Systematic Review and Meta-analysis. Hum Vaccin Immunother. 2020;16(3):581–9.

31. Vygen-Bonnet S, Hellenbrand W, Garbe E, von Kries R, Bogdan C, Heininger U, et al. Safety and effectiveness of acellular pertussis vaccination during pregnancy: a systematic review. BMC Infect Dis [Internet]. 2020;20(1):136. Available from: http://ovidsp.ovid.com/ovidweb.cgi?T=JS&PAGE=reference&D=med17&NEWS=N&AN=32054444

32. Furuta M, Sin J, Ng ESW, Wang K. Efficacy and safety of pertussis vaccination for pregnant women - a systematic review of randomised controlled trials and observational studies. BMC Pregnancy Childbirth [Internet]. 2017;17(1):390. Available from: http://ovidsp.ovid.com/ovidweb.cgi?T=JS&PAGE=reference&D=med14&NEWS=N&AN=29166874

33. Badell ML, Meaney-Delman D, Tuuli MG, Rasmussen SA, Petersen BW, Sheffield JS, et al. Risks Associated With Smallpox Vaccination in Pregnancy: A Systematic Review and Meta-analysis. Obstetrics and gynecology [Internet]. 2015;125(6):1439–51. Available from: http://ovidsp.ovid.com/ovidweb.cgi?T=JS&PAGE=reference&D=med12&NEWS=N&AN=26000516

34. Nunes MC, Aqil AR, Omer SB, Madhi SA. The Effects of Influenza Vaccination during Pregnancy on Birth Outcomes: A Systematic Review and Meta-Analysis. Am J Perinatol [Internet]. 2016;33(11):1104–14. Available from: http://ovidsp.ovid.com/ovidweb.cgi?T=JS&PAGE=reference&D=med13&NEWS=N&AN=27603545

35. Laris-Gonzalez A, Bernal-Serrano D, Jarde A, Kampmann B. Safety of administering live vaccines during pregnancy: A systematic review and meta-analysis of pregnancy outcomes. Vaccines (Basel) [Internet]. 2020;8(1):124. Available from: https://www.mdpi.com/2076-393X/8/1/124/pdf

36. Jeong S, Jang EJ, Jo J, Jang S. Effects of maternal influenza vaccination on adverse birth outcomes: A systematic review and Bayesian meta-analysis. PLoS One [Internet]. 2019;14(8):e0220910. Available from: http://ovidsp.ovid.com/ovidweb.cgi?T=JS&PAGE=reference&D=med16&NEWS=N&AN=31412058

37. Bonde U, Joergensen JS, Lamont RF, Mogensen O. Is HPV vaccination in pregnancy safe?. Hum Vaccin Immunother [Internet]. 2016;12(8):1960–4. Available from: http://ovidsp.ovid.com/ovidweb.cgi?T=JS&PAGE=reference&D=med13&NEWS=N&AN=27172372

38. Bratton KN, Wardle MT, Orenstein WA, Omer SB. Maternal influenza immunization and birth outcomes of stillbirth and spontaneous abortion: a systematic review and meta-analysis. Clin Infect Dis [Internet]. 2015;60(5):e11-9. Available from: http://ovidsp.ovid.com/ovidweb.cgi?T=JS&PAGE=reference&D=med12&NEWS=N&AN=25409473

39. Porudominsky R, Gotuzzo EH. Yellow fever vaccine and risk of developing serious adverse events: a systematic review. REVISTA PANAMERICANA DE SALUD PUBLICA-PAN AMERICAN JOURNAL OF PUBLIC HEALTH. 2018;42.

40. Sangar VC, Ghongane BB, Mathur G, Chowdhary AS. Safety and adverse events of prophylactic HPV vaccines among healthy women: A systematic review & meta analysis. Int J Pharm Sci Res [Internet]. 2015;6(4):1779–91. Available from: http://ijpsr.com/?action=download_pdf&postid=19755

41. Kessels J, Tarantola A, Salahuddin N, Blumberg L, Knopf L. Rabies post-exposure prophylaxis: A systematic review on abridged vaccination schedules and the effect of changing administration routes during a single course. Vaccine [Internet]. 2019;37 Suppl 1:A107–17. Available from: http://ovidsp.ovid.com/ovidweb.cgi?T=JS&PAGE=reference&D=med16&NEWS=N&AN=30737043

42. Macias Saint-Gerons D, Castro JL, Sola Arnau I, De Mucio B, Aleman A, Arevalo-Rodriguez I, et al. Adverse events associated with the use of recommended vaccines during pregnancy: An overview of systematic reviews. Vaccine [Internet]. 2020; Available from: http://www.elsevier.com/locate/vaccine

43. Lu QC, Zhang TY, Bundhun PK, Chen C. One “misunderstood” health issue: demonstrating and communicating the safety of influenza a vaccination in pregnancy: a systematic review and meta-analysis. BMC Public Health [Internet]. 2021;21(1):703. Available from: http://ovidsp.ovid.com/ovidweb.cgi?T=JS&PAGE=reference&D=emexb&NEWS=N&AN=634786695

44. Gee J, Weinbaum C, Sukumaran L, Markowitz LE. Quadrivalent HPV vaccine safety review and safety monitoring plans for nine-valent HPV vaccine in the United States. Hum Vaccin Immunother [Internet]. 2016;12(6):1406–17. Available from: http://www.tandfonline.com/loi/khvi20

45. Arbyn M, Xu L. Efficacy and safety of prophylactic HPV vaccines. A Cochrane review of randomized trials. Expert Rev Vaccines [Internet]. 2018;17(12):1085–91. Available from: http://ovidsp.ovid.com/ovidweb.cgi?T=JS&PAGE=reference&D=med15&NEWS=N&AN=30495978

46. McMillan M, Porritt K, Kralik D, Marshall H. Influenza vaccination during pregnancy: A systematic review of effectiveness and safety. JBI Database System Rev Implement Rep [Internet]. 2014;12(6):281–381. Available from: http://www.joannabriggslibrary.org/jbilibrary/index.php/jbisrir/article/download/1269/2037

47. Andersen AR, Kolmos SK, Flanagan KL, Benn CS. Systematic review and meta-analysis of the effect of pertussis vaccine in pregnancy on the risk of chorioamnionitis, non-pertussis infectious diseases and other adverse pregnancy outcomes. Vaccine [Internet]. 2021; Available from: http://www.elsevier.com/locate/vaccine

48. Jefferson T, Rivetti A, Di Pietrantonj C, Demicheli V. Vaccines for preventing influenza in healthy children. Cochrane Database of Systematic Reviews [Internet]. 2018;(2). Available from: http://dx.doi.org/10.1002/14651858.CD004879.pub5

49. Fell D, Savitz D, Kramer M, Knight M, Luteijn J, Marshall H, et al. Maternal influenza and pregnancy outcomes-a systematic review of comparative studies. J Perinat Med [Internet]. 2015;43(SUPPL. 1). Available from: http://ovidsp.ovid.com/ovidweb.cgi?T=JS&PAGE=reference&D=emed16&NEWS=N&AN=72185031

50. Kreuzberger N, Hirsch C, Andreas M, Böhm L, Bröckelmann PJ, Di Cristanziano V, et al. Immunity after COVID‐19 vaccination in people with higher risk of compromised immune status: a scoping review. Cochrane Database of Systematic Reviews [Internet]. 2022;(8). Available from: http://dx.doi.org/10.1002/14651858.CD015021

51. Kurasawa K.  AO  - Kurasawa KO https://orcid. org/0000 0003 2224 4455. Maternal vaccination-current status, challenges, and opportunities. Journal of Obstetrics and Gynaecology Research [Internet]. 2022; Available from: http://obgyn.onlinelibrary.wiley.com/hub/journal/10.1111/(ISSN)1447-0756/

52. M. T, C. T, S. S, I. P, G. S, R. C, et al. Effectiveness and safety of COVID-19 vaccine in pregnant women: a systematic review with meta-analysis. BJOG [Internet]. 2022; Available from: http://ovidsp.ovid.com/ovidweb.cgi?T=JS&PAGE=reference&D=emexb&NEWS=N&AN=639659246

53. S.A.S. A, M. M, S. S, P. H, M. D, N. N, et al. Complications of COVID-19 Vaccines during Pregnancy; a Systematic Review. Arch Acad Emerg Med [Internet]. 2022;10(1):e76. Available from: http://journals.sbmu.ac.ir/aaem/index.php/AAEM/issue/archive

54. I. H, M.O. K, K. N, S. M, M. N, S. M, et al. Is it safe and effective to administer COVID-19 vaccines during pregnancy? A systematic review and meta-analysis. Am J Infect Control [Internet]. 2022; Available from: http://www.journals.elsevier.com/ajic-american-journal-of-infection-control/

55. Obaid AF, Shlash AMJ, Abdulrasol ZA, Lafta MA. The consequences of COVID-19 and its vaccine on pregnant and lactating mothers. Egypt J Immunol [Internet]. 2022;29(4):58–74. Available from: http://ovidsp.ovid.com/ovidweb.cgi?T=JS&PAGE=reference&D=med22&NEWS=N&AN=36197154

56. Badell ML, Dude CM, Rasmussen SA, Jamieson DJ. Covid-19 vaccination in pregnancy. BMJ [Internet]. 2022;378:e069741. Available from: http://ovidsp.ovid.com/ovidweb.cgi?T=JS&PAGE=reference&D=med22&NEWS=N&AN=35948352

57. Ignaszak-Kaus N, Chmaj-Wierzchowska K, Wszolekl K, Wilczak M. COVID-19 Vaccination and Pregnancy. Clin Exp Obstet Gynecol. 2022;49(10).

58. Nunes MC, Madhi SA. COVID-19 vaccines in pregnancy. Trends Mol Med. 2022;28(8):662–80.

59. Watanabe A, Yasuhara J, Iwagami M, Miyamoto Y, Yamada Y, Suzuki Y, et al. Peripartum Outcomes Associated With COVID-19 Vaccination During Pregnancy A Systematic Review and Meta-analysis. JAMA Pediatr.

60. Piekos SN, Price ND, Hood L, Hadlock JJ. The impact of maternal SARS-CoV-2 infection and COVID-19 vaccination on maternal-fetal outcomes. Reprod Toxicol [Internet]. 2022;114:33–43. Available from: http://ovidsp.ovid.com/ovidweb.cgi?T=JS&PAGE=reference&D=med22&NEWS=N&AN=36283657

61. Binks MJ, Moberley SA, Balloch A, Leach AJ, Nelson S, Hare KM, et al. PneuMum: Impact from a randomised controlled trial of maternal 23-valent pneumococcal polysaccharide vaccination on middle ear disease amongst Indigenous infants, Northern Territory, Australia. Vaccine [Internet]. 2015;33(48):6579–87. Available from: http://ovidsp.ovid.com/ovidweb.cgi?T=JS&PAGE=reference&D=med12&NEWS=N&AN=26529076

62. McHugh L, Binks M, Ware RS, Snelling T, Nelson S, Nelson J, et al. Birth outcomes in Aboriginal mother-infant pairs from the Northern Territory, Australia, who received 23-valent polysaccharide pneumococcal vaccination during pregnancy, 2006-2011: The PneuMum randomised controlled trial. Aust N Z J Obstet Gynaecol [Internet]. 2020;60(1):82–7. Available from: http://ovidsp.ovid.com/ovidweb.cgi?T=JS&PAGE=reference&D=med17&NEWS=N&AN=31198999

63. Barug D, Pronk I, van Houten MA, Versteegh FGA, Knol MJ, van de Kassteele J, et al. Maternal pertussis vaccination and its effects on the immune response of infants aged up to 12 months in the Netherlands: an open-label, parallel, randomised controlled trial. Lancet Infect Dis [Internet]. 2019;19(4):392–401. Available from: http://ovidsp.ovid.com/ovidweb.cgi?T=JS&PAGE=reference&D=med16&NEWS=N&AN=30938299

64. Tapia MD, Sow SO, Tamboura B, Tégueté I, Pasetti MF, Kodio M, et al. Maternal immunisation with trivalent inactivated influenza vaccine for prevention of influenza in infants in Mali: a prospective, active-controlled, observer-blind, randomised phase 4 trial. Lancet Infect Dis. 2016;16(9):1026–35.

65. Madhi SA, Polack FP, Piedra PA, Munoz FM, Trenholme AA, Simões EAF, et al. Respiratory Syncytial Virus Vaccination during Pregnancy and Effects in Infants. New England Journal of Medicine. 2020;383(5):426–39.

66. Donders GGG, Halperin SA, Devlieger R, Baker S, Forte P, Wittke F, et al. Maternal Immunization With an Investigational Trivalent Group B Streptococcal Vaccine. Obstetrics and Gynecology [Internet]. 2016;127(2):213–21. Available from: http://journals.lww.com/greenjournal

67. Muňoz FM, Swamy GK, Hickman SP, Agrawal S, Piedra PA, Glenn GM, et al. Safety and Immunogenicity of a Respiratory Syncytial Virus Fusion (F) Protein Nanoparticle Vaccine in Healthy Third-Trimester Pregnant Women and Their Infants. Journal of Infectious Diseases. 2019;220(11):1802–15.

68. Steinhoff MC, Katz J, Englund JA, Khatry SK, Shrestha L, Kuypers J, et al. Year-round influenza immunisation during pregnancy in Nepal: a phase 4, randomised, placebo-controlled trial. Lancet Infect Dis. 2017;17(9):981–9.

69. Kozuki N, Katz J, Englund JA, Steinhoff MC, Khatry SK, Shrestha L, et al. Impact of maternal vaccination timing and influenza virus circulation on birth outcomes in rural Nepal. Int J Gynaecol Obstet [Internet]. 2018;140(1):65–72. Available from: http://ovidsp.ovid.com/ovidweb.cgi?T=JS&PAGE=reference&D=med15&NEWS=N&AN=28984909

70. Nunes MC, Cutland CL, Moultrie A, Jones S, Ortiz JR, Neuzil KM, et al. Immunogenicity and safety of different dosing schedules of trivalent inactivated influenza vaccine in pregnant women with HIV: a randomised controlled trial. Hugo A Stoltenkamp LA, Abdoola Y, van Niekerk N, Treurnicht F SP, editor. Lancet HIV [Internet]. 2020;7(2):e91–103. Available from: http://ovidsp.ovid.com/ovidweb.cgi?T=JS&PAGE=reference&D=med17&NEWS=N&AN=31911146

71. Madhi SA, Cutland CL, Jose L, Koen A, Govender N, Wittke F, et al. Safety and immunogenicity of an investigational maternal trivalent group B streptococcus vaccine in healthy women and their infants: a randomised phase 1b/2 trial. Lancet Infect Dis [Internet]. 2016;16(8):923–34. Available from: http://ovidsp.ovid.com/ovidweb.cgi?T=JS&PAGE=reference&D=med13&NEWS=N&AN=27139805

72. Perrett KP, Halperin SA, Nolan T, Martinez Pancorbo C, Tapiero B, Martinon-Torres F, et al. Immunogenicity, transplacental transfer of pertussis antibodies and safety following pertussis immunization during pregnancy: Evidence from a randomized, placebo-controlled trial. Vaccine [Internet]. 2020;38(8):2095–104. Available from: http://ovidsp.ovid.com/ovidweb.cgi?T=JS&PAGE=reference&D=medl&NEWS=N&AN=31776029

73. Swamy GK, Metz TD, Edwards KM, Soper DE, Beigi RH, Campbell JD, et al. Safety and immunogenicity of an investigational maternal trivalent group B streptococcus vaccine in pregnant women and their infants: Results from a randomized placebo-controlled phase II trial. Vaccine [Internet]. 2020;38(44):6930–40. Available from: http://www.elsevier.com/locate/vaccine

74. Munoz FM, Patel SM, Keitel WA, Jackson LA, Swamy GK, Edwards KM, et al. Safety and immunogenicity of three seasonal inactivated influenza vaccines among pregnant women and antibody persistence in their infants. Vaccine [Internet]. 2020;38(33):5355–63. Available from: http://www.elsevier.com/locate/vaccine

75. Hoang HTT, Leuridan E, Maertens K, Nguyen TD, Hens N, Vu NH, et al. Pertussis vaccination during pregnancy in Vietnam: Results of a randomized controlled trial Pertussis vaccination during pregnancy. Vaccine. 2016;34(1):151–9.

76. Kostinov MP, Cherdantsev AP, Akhmatova NK, Praulova DA, Kostinova AM, Akhmatova EA, et al. Immunogenicity and safety of subunit influenza vaccines in pregnant women. ERJ Open Res [Internet]. 2018;4(2). Available from: https://www.cochranelibrary.com/central/doi/10.1002/central/CN-01611245/full

77. Wanlapakorn N, Maertens K, Chaithongwongwatthana S, Srimuan D, Suratannon N, Vongpunsawad S, et al. Assessing the reactogenicity of Tdap vaccine administered during pregnancy and antibodies to Bordetella pertussis antigens in maternal and cord sera of Thai women. Vaccine [Internet]. 2018;36(11):1453–9. Available from: http://ovidsp.ovid.com/ovidweb.cgi?T=JS&PAGE=reference&D=med15&NEWS=N&AN=29426663

78. Vesikari T, Virta M, Heinonen S, Eymin C, Lavis N, Chabanon AL, et al. Immunogenicity and safety of a quadrivalent inactivated influenza vaccine in pregnant women: a randomized, observer-blind trial. Hum Vaccin Immunother [Internet]. 2020;16(3):623–9. Available from: http://www.tandfonline.com/loi/khvi20

79. Simões EAF, Nunes MC, Carosone-Link P, Madimabe R, Ortiz JR, Neuzil KM, et al. Trivalent influenza vaccination randomized control trial of pregnant women and adverse fetal outcomes. Vaccine [Internet]. 2019;37(36):5397‐5403. Available from: https://www.cochranelibrary.com/central/doi/10.1002/central/CN-01964897/full

80. Skipetrova A, Wartel TA, Gailhardou S. Dengue vaccination during pregnancy - An overview of clinical trials data. Vaccine [Internet]. 2018;36(23):3345–50. Available from: http://ovidsp.ovid.com/ovidweb.cgi?T=JS&PAGE=reference&D=med15&NEWS=N&AN=29716774

81. Villarreal Perez JZ, Ramirez Aranda JM, de la O Cavazos M, Zamudio Osuna M de J, Perales Davila J, Ballesteros Elizondo MR, et al. Randomized clinical trial of the safety and immunogenicity of the Tdap vaccine in pregnant Mexican women. Hum Vaccin Immunother [Internet]. 2017;13(1):128–35. Available from: http://ovidsp.ovid.com/ovidweb.cgi?T=JS&PAGE=reference&D=med14&NEWS=N&AN=27686182

82. Halperin SA, Langley JM, Ye L, MacKinnon-Cameron D, Elsherif M, Allen VM, et al. A Randomized Controlled Trial of the Safety and Immunogenicity of Tetanus, Diphtheria, and Acellular Pertussis Vaccine Immunization During Pregnancy and Subsequent Infant Immune Response. Clin Infect Dis [Internet]. 2018;67(7):1063–71. Available from: http://ovidsp.ovid.com/ovidweb.cgi?T=JS&PAGE=reference&D=med15&NEWS=N&AN=30010773

83. Munoz FM, Jackson LA, Swamy GK, Edwards KM, Frey SE, Stephens I, et al. Safety and immunogenicity of seasonal trivalent inactivated influenza vaccines in pregnant women. Vaccine [Internet]. 2018;36(52):8054–61. Available from: http://ovidsp.ovid.com/ovidweb.cgi?T=JS&PAGE=reference&D=med15&NEWS=N&AN=30416018

84. Weinberg A, Muresan P, Laimon L, Pelton SI, Goldblatt D, Canniff J, et al. Safety, immunogenicity, and transplacental antibody transport of conjugated and polysaccharide pneumococcal vaccines administered to pregnant women with HIV: a multicentre randomised controlled trial. 2021 [cited 2023 Jan 30];8. Available from: https://doi.org/10.1016/
